# A possible role for proinflammatory activation via cGAS-STING pathway in atherosclerosis induced by accumulation of DNA double-strand breaks

**DOI:** 10.1038/s41598-023-43848-7

**Published:** 2023-09-30

**Authors:** Chiemi Sakai, Keitaro Ueda, Kohei Goda, Rikuto Fujita, Junji Maeda, Shinya Nakayama, Yusuke Sotomaru, Satoshi Tashiro, Masao Yoshizumi, Takafumi Ishida, Mari Ishida

**Affiliations:** 1 Department of Cardiovascular Physiology and Medicine, Graduate School of Biomedical and Health Sciences, Hiroshima University, 1-2-3 Kasumi, Minami-ku, Hiroshima City, Hiroshima 734-8551 Japan; 2 National Hospital Organization, Higashihiroshima Medical Center, Hiroshima City, Japan; 3 Department of Cardiology, Tsuchiya General Hospital, Hiroshima City, Japan; 4 Department of Cellular Biology, Research Institute for Radiation Biology and Medicine, Hiroshima University, Hiroshima City, Japan; 5 Natural Science Center for Basic Research and Development, Hiroshima University, Hiroshima City, Japan; 6 Department of Cardiovascular Medicine, Fukushima Medical University, Fukushima, Japan

**Keywords:** Cell biology, Cardiology

## Abstract

DNA damage contributes to atherosclerosis. However, causative links between DNA double-strand breaks (DSBs) and atherosclerosis have yet to be established. Here, we investigated the role of DSBs in atherosclerosis using mice and vascular cells deficient in Ku80, a DSB repair protein. After 4 weeks of a high-fat diet, Ku80-deficient apolipoprotein E knockout mice (*Ku80*^+/−^*ApoE*^−/−^) displayed increased plaque size and DSBs in the aorta compared to those of *ApoE*^−/−^ control. In the preatherosclerotic stages (two-week high-fat diet), the plaque size was similar in both the *Ku80*^+/−^*ApoE*^−/−^ and *ApoE*^−/−^ control mice, but the number of DSBs and mRNA levels of inflammatory cytokines such as IL-6 and MCP-1 were significantly increased in the *Ku80*^+/−^*ApoE*^−/−^ aortas. We further investigated molecular links between DSBs and inflammatory responses using vascular smooth muscle cells isolated from *Ku80* wild-type and *Ku80*^+/−^ mice. The *Ku80*^+/−^ cells displayed senescent features and elevated levels of inflammatory cytokine mRNAs. Moreover, the cytosolic DNA-sensing cGAS-STING pathway was activated in the *Ku80*^+/−^ cells. Inhibiting the cGAS-STING pathway reduced IL-6 mRNA level. Notably, interferon regulatory factor 3 (IRF3), a downstream effector of the cGAS-STING pathway, was activated, and the depletion of IRF3 also reduced IL-6 mRNA levels in the *Ku80*^+/−^ cells. Finally, DSBs accumulation in normal cells also activated the cGAS-STING-IRF3 pathway. In addition, cGAS inhibition attenuated DNA damage-induced IL-6 expression and cellular senescence in these cells. These results suggest that DSBs accumulation promoted atherosclerosis by upregulating proinflammatory responses and cellular senescence via the cGAS-STING (-IRF3) pathway.

## Introduction

Accumulating evidence suggests that there are strong links between DNA damage and atherosclerosis^1–4^. For instance, some premature aging syndromes caused by mutations in genes involved in genetic stability and DNA repair, such as Hutchinson-Gilford progeria syndrome and Werner syndrome, are accompanied by the development of atherosclerosis at a young age^5,6^. Multiple forms of DNA damage, including oxidative DNA damage (8-OH-dG) and DNA strand breaks, along with elevated levels of DNA repair enzymes, have been detected in atherosclerotic lesions^2,7,8^. However, the role of DNA damage, whether it is a causative element or a byproduct of atherosclerosis, has yet to be established.

DNA damage can arise from exogenous threats like ionizing radiation and chemotherapeutic agents or endogenous sources like replication stress and reactive oxygen species from cellular metabolism^9^. Among the various forms of DNA damage, double-strand breaks (DSBs) are notorious for their complexity to be repaired and thus contributing to their carcinogenic potential. According to the previous estimation, in normal mammalian cells, approximately 50 DSBs spontaneously occur per cell per day due to endogenous cellular processes^10^. DSBs are initially recognized by the Mre11/Rad50/NBS1 (MRN) complex, which recruits an Ataxia telangiectasia mutated (ATM) protein kinase to the DSB sites. ATM is one of the main regulators of the DNA damage response (DDR) that phosphorylates histone H2AX (γH2AX) at the DSB sites, as well as both p53 and checkpoint kinase 2 to activate the downstream signaling cascades^11^. DSBs can lead to either apoptosis or cellular senescence if unrepaired^12^. There are two major pathways to repair DSBs. One is non-homologous end joining (NHEJ) and the other is homologous recombination. In NHEJ, the broken ends are simply ligated; thus, a DNA template is not required, and NHEJ can take place in any phase of the cell cycle. On the other hand, homologous recombination is a template-dependent repair; therefore, it can only take place in the S–G2 phases of the cell cycle. Cardiovascular cells in adult tissues are thought to be in a quiescent state^13,14^; thus, DSBs in these cells are suspected to be repaired by the NHEJ pathway. Ku80 is an abundant and highly conserved protein, and together with Ku70 forms the Ku heterodimer, which binds to DSB ends and facilitates NHEJ repair^15^. Ku80-deficient cells and mice show hypersensitivity to ionizing radiation, DSB repair defects, and premature aging^16^. Therefore, Ku80-deficient models may be a suitable means to study the role of DSBs accumulation in disease.

Recently, the crosstalk between the DDR and inflammatory response has been revealed. ATM has been reported to activate the transcription factor NF-κB via NF-κB essential modulator (NEMO)^17^. Cellular senescence has been proposed as a link between DNA damage and inflammation due to its feature of secreting a myriad of proinflammatory cytokines, chemokines and growth factors, referred to as senescence-associated secretory phenotype (SASP)^18–20^. Cytoplasmic chromatin fragments generated from DNA damage are reported to activate innate immunity via inflammasome activation, the toll-like receptor 9 (TLR9) pathway or the cyclic GMP-AMP synthase (cGAS)-stimulator of interferon genes (STING) pathway^21–24^. Hence, DNA damage may be associated with the development of atherosclerosis, a chronic inflammatory disease, through these pathways.

In the present study, we investigated the role of DSBs in atherosclerosis development using Ku80-deficient apolipoprotein E-knockout (*ApoE*^−/−^) mice. The *ApoE*^−/−^ mice are prone to atherosclerosis because homozygous deletion of the *apoE* gene causes poor lipoprotein clearance, leading to severe hypercholesterolemia^25^. We examined whether accumulation of DSBs accelerated atherosclerosis development in these mice and whether DNA damage could be a causal factor of atherosclerosis. We further investigated the molecular mechanisms that link DSBs and atherosclerosis by using aortic smooth muscle cells derived from Ku80-deficient mice.

## Materials and Methods

The data that support the findings of this study are available from the corresponding author upon reasonable request.

### Animal experiments

All mouse experiments were approved by the Institutional Animal Care and Use Committees of Hiroshima University. All animal procedures were performed under the guidelines established by the Animal Experimentation Committees of Hiroshima University. All the animal experiments in this study complied with the ARRIVE guidelines. *ApoE*^−/−^ mice on C57BL/6 J background (Stock #002052) were purchased from The Jackson Laboratory (ME, USA). *Ku80*^−/−^ 129/Svj mice were generously provided by Dr. Noboru Motoyama with permission from Dr. André Nussenzweig^26^. The *Ku80*^−/−^ 129/Svj mice were backcrossed into a C57BL/6 J background and then cross-bred with the *ApoE*^−/−^ mice to generate *Ku80*^+/−^*ApoE*^−/−^ mice. Their genotypes were identified by polymerase chain reaction (PCR). The primers used for genotyping were as follows: Ku80 KO, forward, 5′ AGCTTCCACCCTCTAGAGAT 3′; Ku80 wild-type, forward, 5′ TAAAGCGCATGCTCCAGACT 3′; and Ku80 complement, reverse, 5′ ATTGTGATGTGTGGGACACG 3′. The male mice were fed with a high-fat diet (F2HFD1: fat, 36% of total kcal; cholesterol, 1.25%; Oriental Yeast Co., Tokyo, Japan) starting from 60 days of age for either 2 or 4 weeks. All mice were housed under a 12-h light and dark cycle. The mice were asphyxiated by gaseous carbon dioxide, and the aorta was isolated from them.

### Atherosclerotic Lesion analysis

The thoracic aortas isolated from the mice were fixed in 4% paraformaldehyde. Perivascular fat was removed and the aorta was opened longitudinally. Oil red-O staining was performed on the aorta. Images of the aorta were analyzed using ImageJ software^27^ to quantify plaque area.

### Immunohistochemical detection of DNA double-strand breaks in murine aorta

Paraffin-embedded murine aortic arch was cross sectioned. The tissue sections were deparaffinized in xylene. Heat-induced epitope retrieval was performed on these sections at 97 °C for 45 min. Sections were incubated with anti-phosphorylated histone H2AX (γH2AX) (ser139) after avidin/biotin block for 20 min each at room temperature. On the following day, sections were incubated with a biotinylated secondary antibody for 30 min at room temperature. γH2AX-positive cells were visualized by DAB reaction and counterstained with hematoxylin. For quantification, the number of γH2AX-positive cells was counted and divided by the number of total cells in the media and intima.

### Immunofluorescent staining of aortic tissue sections

Paraffin-embedded mice aortic arch was cross sectioned. The tissue sections were deparaffinized in xylene. Heat-induced epitope retrieval was performed on these sections at 97 °C for 45 min. Sections were incubated with blocking buffer (1% BSA in PBST) for 1 h at a room temperature, and then incubated with anti-γH2AX (ser139) antibodies overnight. On the following day sections were incubated with a mixture of secondary antibodies conjugated with Cy3 for 2 h at a room temperature. Sections were counterstained with Hoechst 33342 (Dojindo, Kumamoto, Japan). Fluorescent images were captured with BZ-X700 (KEYENCE, Osaka, Japan).

### Isolation and culture of murine vascular smooth muscle cells

Aortic tissues (from aortic arch to descending thoracic artery) were removed from CO_2_ asphyxiated mice and perivascular fat was removed. The adventitia was removed from the aortas following an incubation with collagenase type II (Worthington, NJ, USA) at 37 °C for 3–5 min in an air incubator. Longitudinally incised aortas were gently wiped with cotton swabs to remove the endothelium and then incubated with a mixture of collagenase type II and elastase (Sigma) for tissue dissociation at 37 °C for up to 45 min. The dissociated aortic tissues were incubated with DMEM supplied with 20% FBS until vascular smooth muscle cells (VSMCs) proliferated. The supply of FBS was reduced to 10% when the cells began to stably proliferate. The cells were passaged at a 1:3 ratio when they reached 100% confluence. Cells at passages 5–8 were used for experiments.

### Real-time quantitative PCR analysis

Cells or aortic tissues were homogenized with TRIzol™ Reagent (ThermoFischer). RNA was extracted according to the manufacturer’s protocol, and reverse-transcribed to cDNA using ReverTra Ace® (TOYOBO, Osaka, Japan). Quantitative PCR was performed on the cDNA samples with StepOnePlus™ real-time system (Applied Biosystems) using THUNDERBIRD® SYBR® qPCR Mix (TOYOBO). The comparative Ct method was used to determine relative mRNA levels. The Ct value of the target gene was normalized to that of 18s ribosomal RNA, an internal control gene, for each sample (∆Ct). The difference between two samples was calculated (∆∆Ct), and then fold change was calculated by the 2^−∆∆Ct^ formula (target gene/18s). Primer sequences are listed in Supplemental Table 1.

### Immunofluorescent staining of VSMCs

Cells plated on cover slips were fixed with 4% paraformaldehyde for 10 min at room temperature, and then permeabilized with PBS containing 0.5% TritonX and 0.1% SDS for 5 min at room temperature. The cells were incubated with a mixture of anti-γH2AX and anti-phosphorylated ATM (ser1981) antibodies for 30 min at 37 °C, then with a mixture of secondary antibodies conjugated with Cy3 and FITC for dual-color staining. The cells were then counterstained with 4′,6-diamidino-2-phenylindole (DAPI). Images were captured with Axioplan 2 Imaging Microscope (Carl Zeiss). γH2AX and pATM foci were counted in at least 100 cells. The numbers of γH2AX and pATM foci were divided by the number of DAPI to calculate the average foci number.

### Neutral comet assay

The neutral comet assay was performed using Single Cell Gel Electrophoresis Assay Kit (TRAVIGEN, Gaitherburg, MD) according to the manufacture’s protocol. Briefly, cells exposed to hydrogen peroxide (50 µM for 2 or 24 h) were trypsinized, mixed with preheated LMAgarose and applied onto CometSlide. The slides were immersed in Lysis Buffer for one hour at 4 °C and then subjected to electrophoresis. The cells were stained with SYBR Gold. Fluorescent images were captured with BZ-X700 (KEYENCE, Osaka, Japan) and analyzed by the ImageJ software (National Institute of Health) using a plugin OpenComet. The extent of DNA damage was quantified as comet tail moment. At least 373 cells were counted for each group.

### Immunoblotting analysis

Cells were lysed in RIPA buffer (20 mM Tris, pH 7.3, 150 mM NaCl, 5 mM EDTA, 25 mM NaF, 25 mM sodium pyrophosphate, 1% TritonX, 0.1% SDS, 0.5% deoxycholate, 10% glycerol), sonicated, and centrifuged at 14,000*g* at 4 °C for 10 min. Protein from aortic tissue was extracted using TRIzol™ Reagent (ThermoFisher) according to Kopec et al.’s^28^ protocol. Protein concentrations were determined by DC™ protein assay (Bio-Rad). The protein samples were separated by SDS-PAGE and transferred to nitrocellulose membranes, which were then incubated with primary antibodies at 4 °C overnight and subsequently with secondary antibodies at room temperature for an hour. Protein bands were detected by Western Lighting Plus-ECL (PerkinElmer) using X-ray films. Antibodies are listed in Supplemental Table 2.

### Gene silencing

Cells were transfected with short interfering RNA (siRNA) against mouse *Mb21d1*(*Cgas*) (Mm_E330013A19Rik_3 or 5, QIAGEN), mouse *Tmem173* (*Sting*) (s91057 or s91058, ambion®, ThermoFisher), mouse *Irf3* (s79432, ambion®, ThermoFisher), or a negative control cocktail (AllStars Neg. Control siRNA, QIAGEN) using Lipofectamine™ RNAiMAX Transfection Reagent (ThermoFisher) 48–72 h prior to harvesting samples.

### Senescence-associated beta-galactosidase (SA β-gal) assay

SA β-gal activity in cells was detected with SPiDER-βgal from Dojindo Laboratories (Kumamoto, Japan) according to the protocol specified by the manufacturer. In brief, cells in a 96-well plate were washed with HBSS, incubated with Bafilomycin A1 working solution for 1 h, and then incubated with SPiDER-βgal working solution at 37 °C for 1 h. The cells were fixed with 4% paraformaldehyde for 10 min at room temperature, stained with Phalloidin-iFluor™ 555 Conjugate (Cayman Chemical, MI, USA) for 1 h, and then counterstained with DAPI. Images were captured (Excitation: 488 nm, Emission: 545 nm) and assessed using the Opera Phenix™ screening system (PerkinElmer, MA, USA). Cells with green fluorescence in the cytoplasm were identified as SA β-gal positive.

### Quantification of cytosolic DNA

For the quantification of cells with cytosolic DNA, cells were stained with DAPI. Extranuclear DNA stained with DAPI were counted. Cells with cytosolic DNA was calculated by dividing the number of extranuclear DNA by the number of nuclei. For the quantification of cytosolic DNA of nuclear origin, cytosolic nuclear DNA was isolated and quantified according to the previously described method^29^. Briefly, trypsinized cells were aliquoted into two tubes and centrifuged at 1000 rpm for 3 min. Cell pellet in one tube was resuspended in 200 µl of 50 µM NaOH and boiled for 30 min to solubilize DNA from whole cell. Cell pellet in the other tube was resuspended in 200 µl of digitonin buffer (150 mM NaCl, 50 mM HEPES pH 7.4, and 25 µg/mL digitonin), incubated on a tube rotator at 4 ºC for 10 min, and then centrifuged at 17,000g for 10 min. The supernatants were cytosolic extracts free of nuclear, mitochondrial and endoplasmic reticulum contamination. DNA was isolated from the cytosolic extracts using QIAQuick Nucleotide Removal Columns (QIAGEN). DNA samples from both whole cells and the cytosolic extracts were subjected to quantitative PCR. Primers for β2 microglobulin were used to detect nuclear DNA. The CT values of DNA from whole cells served as normalization controls for the CT values of DNA from the cytosolic extracts. The primer sequences are shown in Supplemental Table 1.

### ELISA of 2′3′-cGAMP

Cells treated with hydrogen peroxide (100 µM) were cultured for 3 days. Cell lysates were then collected using M-PER® Mammalian Protein Extraction Reagent (Thermo Scientific). Measurement of the 2′3′-cyclic GMP-AMP (cGAMP) concentrations in cell lysates was conducted using a 2′3′-cGAMP ELISA kit (Cayman chemical, MI, USA) according to the manufacturer’s protocol. The 96-well plate included with the kit was read at a wavelength of 450 nm with Varioskan Flash (Thermo Scientific).

### IRF3 nuclear translocation

Nuclear IRF3 was detected by immunofluorescent staining. Images were captured with BZ-X700 (KEYENCE, Osaka, Japan). Cells with nuclear IRF3 were counted and the number was divided by the number of nuclei to determine the percentage. At least 120 cells were counted. IRF3 antibody specificity was confirmed by performing immunofluorescent staining in *Irf3*-silenced cells (Supplemental Figure 4D).

### H_2_O_2_-induced cytosolic DNA and cellular senescence in VSMCs

Cells were exposed to hydrogen peroxide (100 µM) once and incubated for 3 days without changing media to induce cytosolic DNA or 7 days with changing media on the third day from hydrogen peroxide exposure to induce cellular senescence, then samples were collected.

### Statistical analysis

The data are expressed as mean ± SEM, and were collected from at least three independent experiments. Statistical significance between two groups was determined by unpaired Student’s *t*-test, or the Mann–Whitney *U* test, and differences among three or more groups were determined by one-way analysis of variance followed by Tukey’s multiple comparison test. *P* values less than 0.05 were considered statistically significant. RStudio software (Vienna, Austria. URL https://www.R-project.org/.) was used for statistical analysis. Graph plots were created by GraphPad Prism 9 software.

## Results

### Atherosclerosis and DSBs were augmented in Ku80-deficient mice.

**Figure 1:**
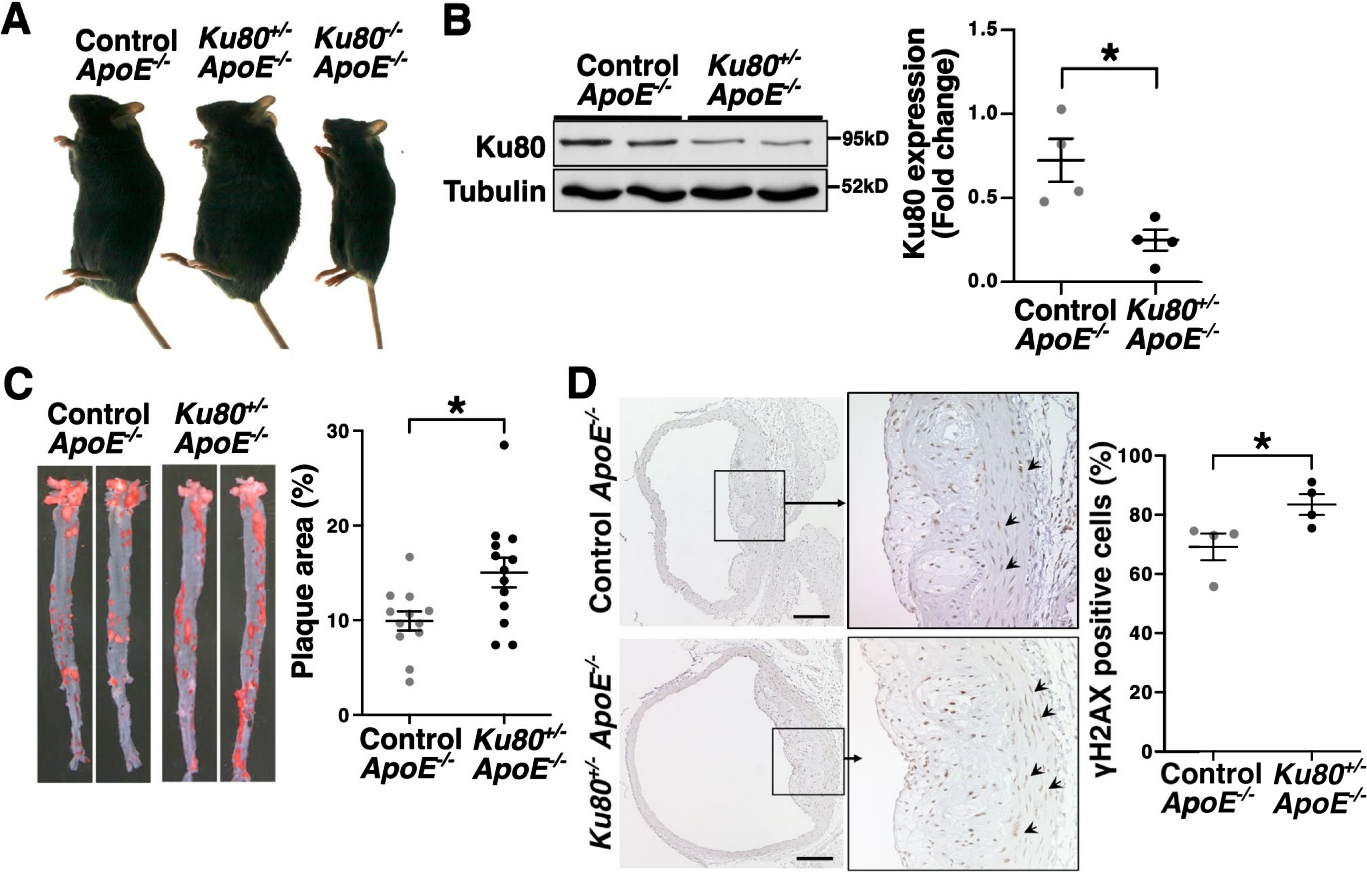
Ku80 deficiency enhanced atherosclerosis and DNA double-stand break (DSB) accumulation. (**A**) Representative images of male mice at 140 days of age (**B**) Ku80 protein expression in the aortas of *ApoE*^−/−^control and *ApoE*^−/−^*Ku80*^+/−^ mice. Bands are cropped from the original blots presented in Supplemental Figure S7. Quantification of Ku80 protein on the right (control; n = 4, *Ku80*^+/−^; n = 4). *p < 0.05. (**C**) Oil Red O staining of the aortas of *ApoE*^−/−^control and *ApoE*^−/−^*Ku80*^+/−^ mice fed on a high-fat diet for 4 weeks starting from 60 days of age. Images of the aortas on left. Quantification of atherosclerotic plaque area on right (control; n = 12, *Ku80*^+/−^; n = 13). (**D**) DNA double-strand breaks in the aortas were stained with anti-phosphorylated Histone H2AX (γH2AX) antibodies, a marker of DNA double-strand breaks. The aortas were obtained from mice fed on a high-fat diet for 4 weeks. γH2AX positive cells (in brown) were indicated by the arrows. The nuclei were stained with hematoxylin (in blue). Arrowheads indicate γH2AX positive cells. Bar = 100 µm. Quantification of γH2AX positive cells on the right (control; n = 4, *Ku80*^+/−^; n = 4). *p < 0.05. Statistical significance between groups was determined using unpaired Student’s *t*-test. Data are expressed as mean ± SEM.

In the present study, Ku80 heterozygous knockout (*Ku80*^+/−^) mice were used as a means to investigate roles of DSBs accumulation in atherosclerosis. Although Ku80 homozygous knockout (*Ku80*^−/−^) mice are viable, they are rarely born and present severe growth retardation (Fig. 1A)^26^. In the *Ku80*^+/−^ mice, the protein expression of Ku80 was less than half the level of wild type mice (Fig. 1B). There were no significant differences in the Ku80 protein levels between BL/6 J and *ApoE*^−/−^ controls (Supplemental Figure 1). To investigate the impact of DSBs accumulation on atherosclerosis, we generated *Ku80*^+/−^* ApoE*^−/−^ mice and fed them a high-fat diet for 4 weeks, starting from 60 days of age. There were no significant differences in plasma characteristics after 4 weeks of a high-fat diet between the *Ku80*^+/−^* ApoE*^−/−^ mice and *ApoE*^−/−^ controls (Table 1). Oil red-O staining showed that plaque area was significantly increased in the *Ku80*^+/−^* ApoE*^−/−^ mice compared to the *ApoE*^−/−^ controls (Fig. 1C). The aortic sections were immunostained with antibodies against phosphorylated histone H2AX (γH2AX) to detect DSBs. We found that DSBs were significantly increased in the *Ku80*^+/−^* ApoE*^−/−^ aortas compared with those of the *ApoE*^−/−^ controls (Fig. 1D). These results suggest that accelerated atherosclerosis is accompanied by DSBs accumulation in Ku80-deficient mice.

**Table1:** Plasma characteristics of mice fed on a high-fat diet for 4 weeks.

	Control *ApoE*^−/−^	*Ku80*^+/−^*ApoE*^−/−^	*p* value
Body weight (g)	21.9 ± 4.5	21.7 ± 4.9	0.86
Total cholesterol (mg/dL)	2633.0 ± 77.0	2150.8 ± 191.2	0.13
HDL cholesterol (mg/dL)	15.0 ± 4.5	14.6 ± 2.2	0.61
LDL cholesterol (mg/dL)	799.3 ± 21.2	650.2 ± 60.5	0.15
Blood glucose (mg/dL)	131.4 ± 14.5	149.3 ± 13.5	0.39

The data are expressed as mean ± SEM. Mann–Whitney *U* test was performed.

*HDL* high density lipoprotein; *LDL* low density lipoprotein. Control *ApoE*^−/−^ (n = 14); *Ku80*^+/−^* ApoE*^−/−^ (n = 20).

### DSBs accumulation and proinflammatory cytokine expression were enhanced in Ku80-deficient mice at preatherosclerotic stages.

**Figure 2:**
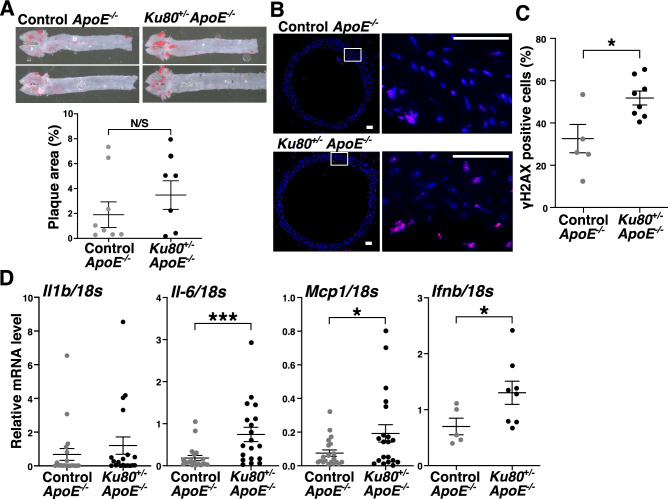
Ku80 deficiency increased DSBs accumulation and proinflammatory cytokine expression in preatherosclerotic stages. (**A**) Representative images of Oil Red O staining of the aortas of mice fed on a high-fat diet for 2 weeks starting from 60 days of age (top). Quantification of atherosclerotic plaque area (bottom) (control; n = 8, *Ku80*^+/−^; n = 7). (**B**) Immunofluorescent images of aortic arch. The aortic sections were stained for γH2AX. γH2AX-positive cells are in red. Nuclei were stained with Hoechst 33342 in blue. Higher magnification of the square area on the right. Bar = 50 µm. (**C**) Quantification of γH2AX-positive cells (Control; n = 5, *Ku80*^+/−^; n = 8). *p < 0.05. (**D**) mRNA levels of proinflammatory cytokines in the aorta were determined by real-time quantitative PCR (qPCR) (n = 5–20 per group). *p < 0.05, ***p < 0.001. Statistical significance between groups was determined using unpaired Student’s *t*-test. Data are expressed as mean ± SEM.

In order to clarify a causative role of DSBs in atherosclerosis, we shortened the high-fat diet period to 2 weeks. As shown in the images of aortas stained with oil red-O, atherosclerotic development was premature and comparable in both the *Ku80*^+/−^*ApoE*^−/−^ mice and *ApoE*^−/−^ mice after 2 weeks of a high-fat diet (Fig. 2A). However, the accumulation of DSBs was more advanced in the *Ku80*^+/−^*ApoE*^−/−^ aortas than in the *ApoE*^−/−^ controls (Fig. 2B,C, and Supplemental Figure 2A). Of note, the mRNA levels of proinflammatory cytokines such as interleukin 6 (IL-6), monocyte chemotactic protein 1 (MCP1) and interferon beta 1 (IFNβ1) in the descending thoracic aorta where atherosclerotic lesions were hardly prominent were significantly elevated in the *Ku80*^+/−^*ApoE*^−/−^ mice compared to the *ApoE*^−/−^ mice (Fig. 2D). Likewise, serum IL-6 level was elevated in the *Ku80*^+/−^*ApoE*^−/−^ mice (Supplemental Figure 2B). Considering that DSBs accumulation and proinflammatory activation have preceded the formation of atherosclerotic plaque, we speculated that impaired DDR caused accumulation of DSBs and induced proinflammatory activation, which accelerated the development of atherosclerosis.

### Ku80-deficient vascular smooth muscle cells exhibited persistent DNA damage response and senescent features

**Figure 3:**
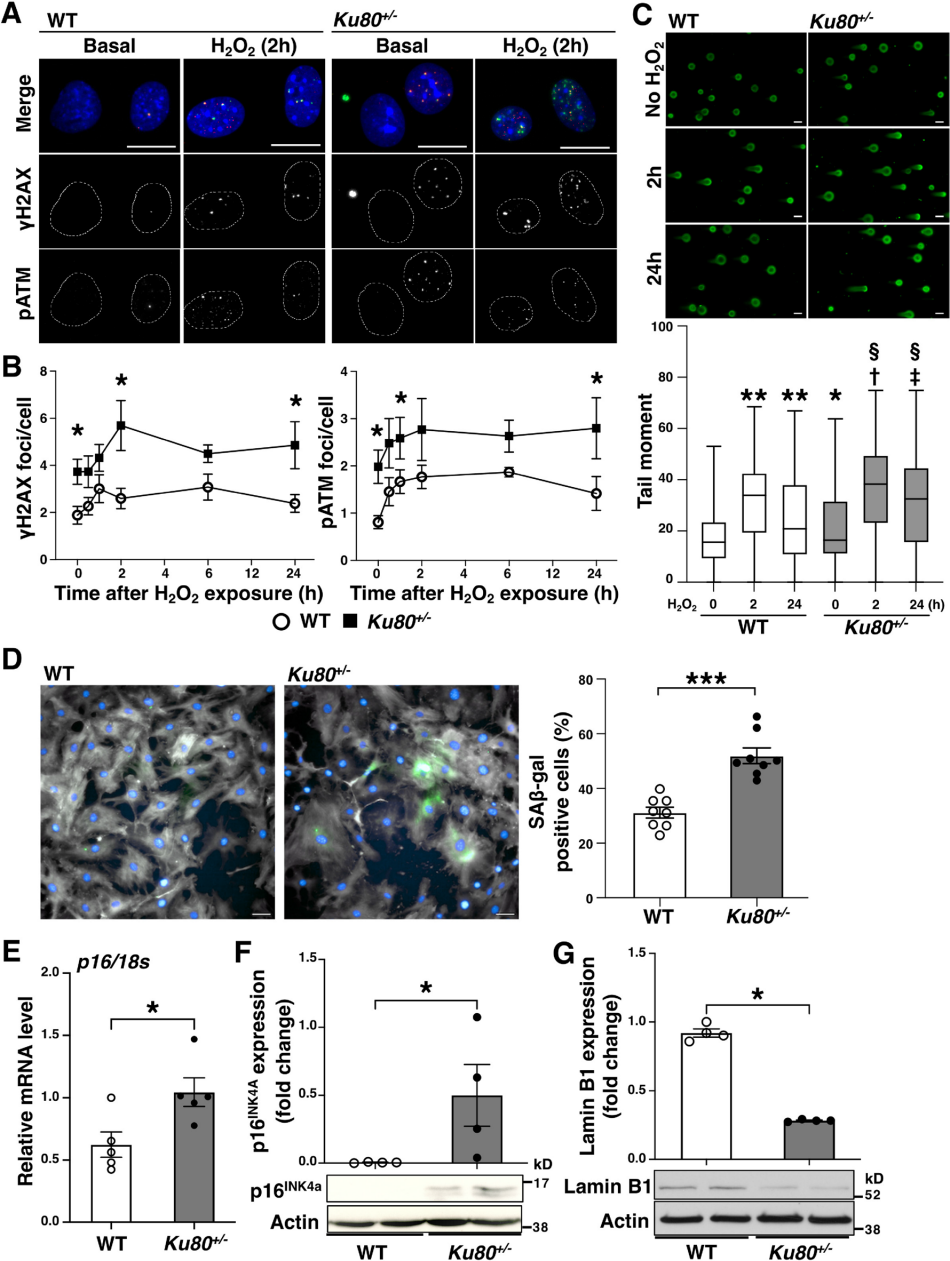
Ku80-deficient vascular smooth muscle cells showed persistent DNA damage response and senescent characteristics. (**A** and **B**) Immunofluorescent analysis of DNA damage response in the wild type (WT) and *Ku80*^+/−^ VSMCs. (**A**) Representative images of cells stained with anti-γH2AX antibody (in green) and anti-phosphorylated ataxia telangiectasia mutated (pATM) antibody (in red) indicating activation of the DNA damage response. Cells were counterstained with DAPI (in blue). Bar = 20 µm. (**B**) Time-course analysis of DSBs accumulation and activation of the DNA damage response. The cells were exposed to hydrogen peroxide (10 µM) for 30 min, 1, 2, 6, or 24 h (n = 7). At least one hundred cells were counted for γH2AX and pATM foci from each group. *p < 0.05. (**C**) Neutral comet assay of WT and *Ku80*^+/−^ VSMCs exposed to hydrogen peroxide (50 µM) for 2 or 24 h. Representative images on the top. Quantification on the bottom. Error bars indicate the median and max/min of at least 373 cells for each group. *p < 0.05, **p < 0.0001 versus WT(no H_2_O_2_); † *p* < 0.0001 versus WT(2 h); ‡ *p* < 0.0001 versus WT(24 h); §*p* < 0.0001 versus *Ku80*^+/−^ (no H_2_O_2_). Statistical significance was determined using one-way ANOVA. (**D**) Staining of senescence-associated β-gal (SA β-gal) in VSMCs. Representative images on the left (WT) and middle (*Ku80*^+/−^) panels (SA β-gal in green, Phalloidin in gray, DAPI in blue). Bar = 50 µm. Quantification of SA β-gal positive cells in percentages on the right panel (n = 8). ***p < 0.001. E) p16 mRNA level (a senescent marker) in cells was quantified by real-time qPCR analysis (n = 5). *p < 0.05. (**F**) Western blot analyses of p16^INK4A^ and (**G**) lamin B1 in cells (n = 4). Bands are cropped from the original blots presented in Supplemental Figure 8. *p < 0.05. Statistical significance between groups was determined using the Mann–Whitney *U* test. Data are expressed as mean ± SEM.

We observed that DSBs accumulation was already present in VSMCs of the mice fed with a high-fat diet for 2 weeks (preatherosclerotic stages) (Fig. 2B and Supplemental Figure 2C). To examine whether the accumulation of DSBs triggers inflammation, we isolated VSMCs from *Ku80*^+/−^ and *Ku80*^+*/*+^ (WT) mice and performed analyses using these primary cell cultures. Based on recent evidence, VSMCs seem to drive the inflammatory process of atherosclerosis^30–32^. We confirmed that the isolated cells were VSMCs by immunocytochemistry, using antibodies against the VSMC specific isoform of alpha-actin (Supplemental Figure 2A)^33–35^. We performed a time course analysis to determine the effect of Ku80 deficiency on DSB formation and DDR activation in response to hydrogen peroxide exposure. We have previously reported that hydrogen peroxide-induced DSBs in human VSMCs^36^. In addition, ApoE-deficient cells seem to be high in the hydrogen peroxide level due to increased NADPH oxidase-derived superoxide production^37^. Immunofluorescent staining showed that DSBs were significantly accumulated in *Ku80*^+/−^ VSMCs under basal conditions. (Fig. 3A,B). Similarly, ATM, which is known to be recruited and activated by the MRN complex in response to DSBs, was phosphorylated at the sites of DSBs in the *Ku80*^+/−^ cells (Fig. 3A,B). We confirmed the phosphorylated-ATM foci were indeed specific to phosphorylated ATM in *Atm*-silenced cells (Supplemental Figure 3B). DSB formation was more enhanced in the *Ku80*^+/−^ cells than in the WT cells at any given time point (Fig. 3B). The WT cells showed a decrease in ATM activation after 24 h of exposure with a peak at 2 h, while the *Ku80*^+/−^ cells showed persistent activation of ATM after 24 h (Fig. 3B). Results from the neutral comet assay were consistent with those from the immunofluorescent analysis of DSBs (Fig. 3C). These results suggest that DDR activation was sustained by the accumulation of DSBs in the *Ku80*^+/−^ cells. Prolonged activation of DDR triggers senescence-associated cell cycle arrest^38^. Indeed, cellular senescence is characterized by excessive DNA damage accumulation^39^. Thus, we assessed other senescence biomarkers. Senescence-associated beta-galactosidase (SA β-gal) activity was significantly increased in the *Ku80*^+/−^ cells (Fig. 3D). Both the mRNA and protein levels of p16^INK4A^ were significantly elevated in the *Ku80*^+/−^ cells under basal condition (Fig. 3E,F). p16^INK4A^-positive VSMCs were detected in atherosclerotic aorta of the *Ku80*^+/−^*ApoE*^−/−^ mice (Supplemental Figure 2D). In addition, the *Ku80*^+/−^ cells showed reduced levels of the nuclear lamina protein lamin B1 (Fig. 3G), also known as a hallmark of senescent cells^19^. Thus, Ku80-deficient VSMCs displayed enhanced DSBs accumulation, persistent DDR and upregulation of senescent markers.

### Ku80 deficiency enhanced proinflammatory cytokine expression in VSMCs

**Figure 4:**
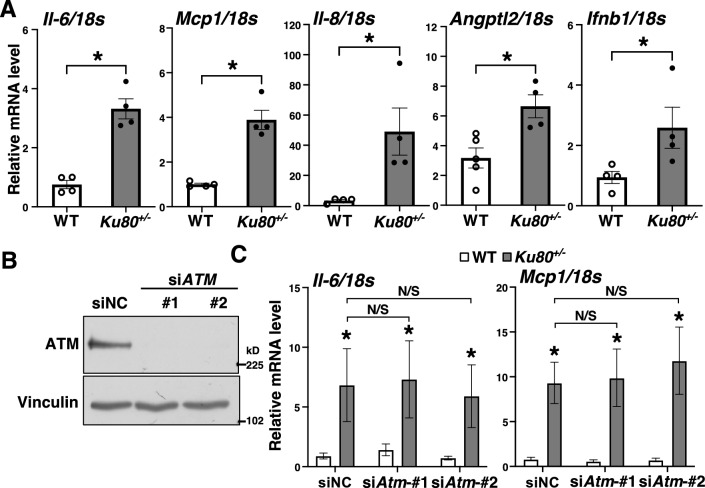
Proinflammatory cytokine levels were elevated in Ku80-deficient VSMCs. (**A**) mRNA levels of proinflammatory cytokines in cells were analyzed by real-time qPCR (n = 4). (**B**) Western blot analysis of ATM in *Atm*-silenced VSMCs. Both WT and *Ku80*^+/−^ cells were transfected with either negative control siRNA (siNC) or siRNA against *Atm*. Bands are cropped from the original blots presented in Supplemental Figure 9. (**C**) mRNA levels of IL-6 and MCP1 in *Atm*-silenced VSMCs were determined by real-time qPCR. *p < 0.05 compared with corresponding WT cells (n = 6). Statistical significance between groups was determined using the Mann–Whitney *U* test. Data are expressed as mean ± SEM.

We next analyzed the mRNA levels of proinflammatory cytokines in *Ku80*^+/−^ and WT VSMCs and found that the mRNA levels of IL-6, MCP1, IL-8, IFNβ1, and angiopoietin like protein 2 (ANGPTL2) were significantly increased in the *Ku80*^+/−^ cells (Fig. 4A) Similarly, levels of secreted IL-6 were elevated in these cells (Supplemental Figure 2B). We suspected that activation of ATM induced proinflammatory responses via activation of NF-κB^17^. However, silencing the ATM gene in *Ku80*^+/−^ and WT VSMCs showed no effect on the mRNA levels of proinflammatory cytokines (Fig. 4B,4C).

### The DNA sensing cGAS-STING pathway was activated in Ku80-deficient VSMCs

**Figure 5:**
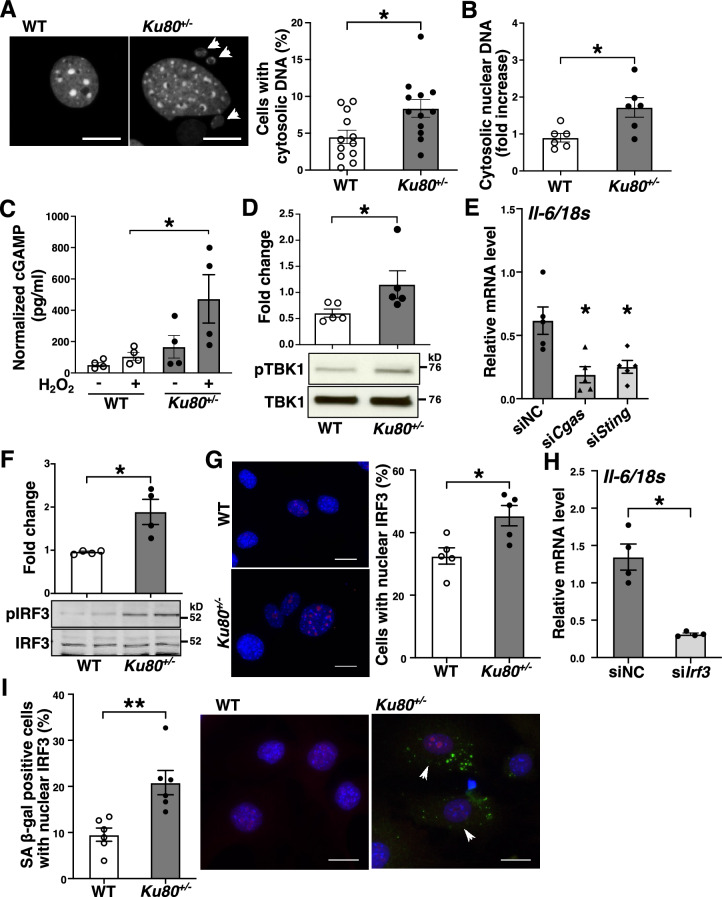
cGAS-STING pathway was upregulated in Ku80-deficient cells. (**A**) VSMCs were stained with DAPI. The left panels are representative images of nuclei. The white arrowheads indicate cytosolic DNA. Bar = 20 µm. Quantification of cells with cytosolic DNA in percentage on the right panel (n = 12). *p < 0.05. (**B**) Cytosolic nuclear DNA in WT and *Ku80*^+/−^ VSMCs was analyzed by real-time qPCR using primers for β2 microglobulin (n = 6). *p < 0.05. (**C**) ELISA assay of 2′3’-cGAMP concentration in cells at basal conditions or treated with hydrogen peroxide (100 µM) for 3 days (n = 4). (**D**) Western blot analyses of phosphorylated TBK1 (Ser172) in whole cell lysates (n = 5), (**E**) *Cgas* or *Sting* was silenced in *Ku80*^+/−^ cells. IL-6 mRNA level was determined by real-time qPCR analysis. *p < 0.05 compared with negative control siRNA (siNC) (n = 5). (**F**) Western blot analysis of phosphorylated IRF3 (Ser396) in whole cell lysates (n = 4). (**G**) IRF3 nuclear translocation was detected by immunofluorescent staining. Representative images on the left panels. Bar = 20 µm. Quantification on the right panel (n = 5). H) IL-6 mRNA levels in *Irf3*-silenced *Ku80*^+/−^ cells. *p < 0.05 compared with negative control siRNA (siNC) (n = 4). (**I**) Quantification of SA β-gal positive cells with nuclear IRF3 (n = 6). Representative images on the middle and right panels. SA β-gal stained in green; IRF3 in red; the nuclei in blue. Arrowheads indicate SA β-gal/nuclear IRF3-positive cells. Bar = 20 µm. **p < 0.01. Bands are cropped from the original blots presented in Supplemental Figure 10. Statistical significance between groups was determined using the Mann–Whitney *U* test. Data are expressed as mean ± SEM.

We investigated the molecular mechanisms by which the accumulation of DSBs activates inflammatory responses. Several groups have reported that DSB fragments are expelled out to the cytosol^40–42^. We also observed a significant increase of cytosolic DNA, particularly of nuclear origin, in *Ku80*^+/−^ VSMCs (Fig. 5A,B). DNA fragments in the cytosol serve as a damage-associated molecular pattern which triggers type I interferon production, such as IFNα and IFNβ^43,44^. The cGAS-STING axis is a DNA sensing pathway known to be activated by self-DNA in the cytosol that induces a type I interferon response^45^. Since IFNβ1 mRNA level was significantly upregulated in the aorta of the *Ku80*^+/−^*ApoE*^−/−^ mice (Fig. 2D) and cultured *Ku80*^+/−^ VSMCs compared to their controls (Fig. 4A), we tested whether the cGAS-STING axis was activated in the *Ku80*^+/−^ cells by assessing cyclic GMP-AMP (cGAMP), a second messenger of cGAS, and TANK-binding kinase 1 (TBK1) phosphorylation, a downstream effector kinase of STING^46^. The *Ku80*^+/−^ cells showed a tendency for higher cGAMP concentration than that of the WT cells under basal conditions and a significant increase under H_2_O_2_-exposed conditions (Fig. 5C). At basal conditions, the *Ku80*^+/−^ cells showed a significant increase in TBK1 phosphorylation (Fig. 5D). To assure that DSB-induced cGAS-STING activation promoted proinflammatory activation, we silenced either the cGAS or STING genes in the *Ku80*^+/−^ cells with siRNA (Supplemental Figure. 4A and 4B). This silencing decreased the mRNA level of IL-6, but not of IFNβ1 or MCP1 (Fig. 5E and Supplemental Figure 4C). The cGAS-STING signaling is mediated by downstream effectors, such as interferon regulatory factor 3 (IRF3) and NF-κB, resulting in subsequent proinflammatory cytokine expression^45^. Both IRF3 and NF-κB were significantly activated in the *Ku80*^+/−^ cells (Fig. 5F,G, Supplemental Figure 5A and 5B). We further examined whether IRF3 or NF-κB participated in upregulation of IL-6 in the *Ku80*^+/−^ cells by silencing these genes (Supplemental Figure 4E and 5C). Surprisingly, transient silencing of *Irf3*, but not of *Rela* (NF-κB p65), attenuated mRNA levels of IL-6 in the *Ku80*^+/−^ cells (Fig. 5H and Supplemental Figure. 5D). We further tested whether cellular senescence and cGAS-STING-IRF3 activation occur coincidently. Cellular senescence was accompanied with cGAS-STING-IRF3 activation more significantly in the *Ku80*^+/−^ cells (Fig. 5I). TBK1 and IRF3 were also activated in the aortic tissues of the *Ku80*^+/−^* ApoE*^−/−^ mice fed with a high-fat diet for 2 weeks (Supplemental Figure 6A and 6B).

### DSBs accumulation promotes cGAS-STING-IRF3 activation and cellular senescence in normal cells

**Figure 6:**
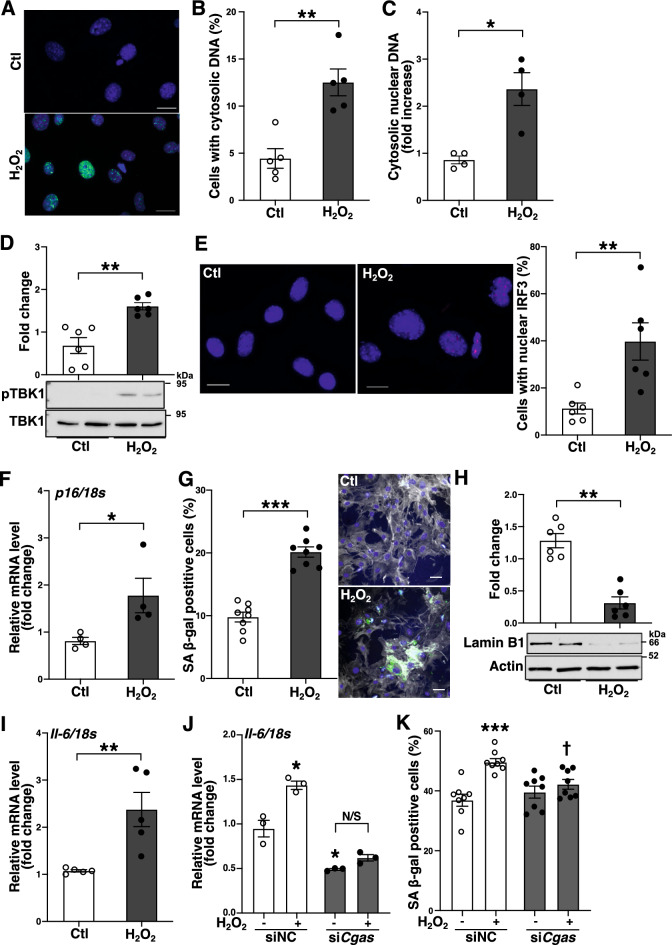
DNA damage induced the cGAS-STING-IRF3 activation and cellular senescence. (**A**) DSB formation in Murine VSMCs after 1-h exposure to hydrogen peroxide (100 µM). γH2AX stained in green; DAPI in blue. Bar = 20 µm. (**B**, **C**) The cells were exposed to hydrogen peroxide (100 µm) for 3 days to induce cytosolic DNA. (**B**)The cells were stained with DAPI and cells with cytosolic DNA were quantified in percentage (n = 5). (**C**) Cytosolic nuclear DNA in the cells was analyzed by real-time qPCR using primers for β2 microglobulin (n = 4). (**D**–**I**) Cellular senescence was induced in murine VSMCs with hydrogen peroxide (100 µM) for 7 days. (**D**) Western blot analysis of phosphorylated TBK1 (Ser172) in whole cell lysates (n = 6). (**E**) IRF3 nuclear translocation was analyzed by immunofluorescent staining. Representative images on the left and middle panels. IRF3 in red; the nuclei stained with DAPI in blue. Bar = 20 µm. Quantification of cells with nuclear IRF3 in percentage on the right panel (n = 6). (**F**) mRNA level of p16 was determined by real-time qPCR analysis (n = 4). (**G**) SA β-gal staining in the cells. Quantification of SA β-gal positive cells in percentage on the left panel (n = 8). Representative images on the right panels (SA β-gal in green, Phalloidin in gray, DAPI in blue). Bar = 50 µm. (**H**) Western blot analysis of lamin B1 in whole cell lysates (n = 6). (**I**) mRNA levels of IL-6 in the cells were determined by real-time qPCR (n = 5). (**J**) The cells were transfected either with negative control siRNA (siNC) or siRNA against *Cgas* (si*Cgas*) 48 h prior to H_2_O_2_ exposure, and then incubated for 3 days after the exposure and IL-6 mRNA levels were determined (n = 3). (**K**) SA β-gal positive rate was determined in the cells transfected either with siNC or si*Cgas* and exposed to H_2_O_2_ for 5 days (n = 8).*p < 0.05, **p < 0.01, ***p < 0.001 compared with control or siNC (no H_2_O_2_), † *p* < 0.05 compared with siNC with H_2_O_2_. N/S: not significant. Statistical significance was determined using the Mann–Whitney U test, Student *t*-test for I, or one-way ANOVA for (**J** and **K**). Data are expressed as mean ± SEM. Bands are cropped from the original blots presented in Supplemental Figure 11.

We examined whether the cellular responses induced by accumulation of DSBs observed in the *Ku80*^+/−^ cells occur in normal cells. DSBs were induced by hydrogen peroxide in murine VSMCs (Fig. 6A). The cells exposed to hydrogen peroxide showed an increase in cytosolic nuclear DNA (Fig. 6B,C). Further, the cells showed TBK1 phosphorylation and an increased level of nuclear IRF3 (Fig. 6D,E) indicating activation of the cGAS-STING pathway followed by IRF3 activation. Concurrently, the cells exhibited senescence features such as increases in p16 mRNA and SA β-gal activity and reduced expression of lamin B1 (Fig. 6F–H). Similar to the *Ku80*^+/−^ cells, the mRNA levels of IL-6 were elevated in the cells (Fig. 6I), which was abrogated by *cGAS* silencing (Fig. 6J). Finally, the increase in SA β-gal-positive cells was also canceled by *cGAS* silencing (Fig. 6K). Together, these data indicate that the accumulation of DSBs increases cytosolic DNA levels, activating cGAS-STING-IRF3 axis which plays a crucial role in cellular senescence and IL-6 expression.

## Discussion

The major findings of this study are that atherosclerosis and DSBs accumulation are augmented in *Ku80*^+/−^*ApoE*^−/−^ mice, and that DSBs accumulation and upregulation of proinflammatory cytokines precede the onset of atherosclerosis in the *Ku80*^+/−^*ApoE*^−/−^ mice. Ku80-deficient VSMCs exhibit persistent DDR, cellular senescence, and upregulated proinflammatory cytokine expression. It is of interest that cytosolic DNA fragments are increased in the Ku80-deficeint VSMCs. In addition, the cGAS-STING-IRF3 axis is activated in these cells. These findings suggest that DSBs play a causative role in atherosclerosis, at least in part, through cytosolic DNA-induced activation of the cGAS-STING pathway, cellular senescence, and the resultant proinflammatory response.

Innate immune DNA-sensing pathways, such as cGAS-STING, TLR9, and inflammasome, are well characterized as key regulators of antiviral host immune defense and autoimmune diseases. Recent reports show the association between these DNA sensors and atherosclerosis. For instance, absent in melanoma 2 (AIM2) inflammasome was reported to regulate atherosclerotic plaque vulnerability^47^. Fukuda et al. demonstrated that a circulating level of “cell-free DNA” was elevated in angiotensin II treated *ApoE*^−/−^ mice, which promoted proinflammatory response via the activation of TLR9^48^. Although we could detect neither TLR9 nor inflammasome activation in Ku80-deficeint VSMCs (data not shown), we observed marked increases of cytosolic DNA fragments (Fig. 5A,B), cGAMP concentration (Fig. 5C), TBK1 phosphorylation (Fig. 5D), and proinflammatory cytokine expression in Ku80-deficient VSMCs (Fig. 4A), which indicate that the accumulation of DSBs and resultant cytosolic DNA fragments induced cGAS-STING activation and subsequent inflammatory responses, thus contributing to atherosclerosis development. Originally, the cGAS-STING pathway was identified as a pathogenic DNA sensor, by which innate immune responses are activated^49^. Upon DNA sensing, cGAS produces the second messenger cGAMP to activate STING, which mediates inflammatory responses via phosphorylation of the inhibitor of kappa B kinase (IKK) and TBK1, and subsequent activation of NF-κB and IRF3, respectively^46^. Interestingly, although IL-6 mRNA levels in the Ku80-deficient cells were mitigated by *Cgas* or *Sting* silencing, IFNβ1 and MCP1 mRNA levels were either not affected or rather increased by the gene silencing (Fig. 5E, Supplemental Figure 4C). It is possible that the expression of IFNβ1 and MCP1 mRNA induced by the accumulation of DSBs and/or resultant cytosolic DNA fragments may require other signal events in addition to GAS-STING in VSMCs. Another interesting finding was that the cGAS-STING-IRF3 axis was predominant in upregulation of IL-6, but not IFNβ1 in VSMCs. Very recently, Fukuda’s group also reported that STING contributes to atherogenesis by activating macrophage-mediated proinflammatory responses^50^. In their report, STING inhibition attenuated NF-κB-mediated inflammation. The inconsistencies between our results and theirs may be explained by differences in the types of cells, stress applied and approaches used to inhibit cGAS or STING. Our findings may be a cell-specific response to DSBs accumulation in VSMCs. Future studies should assess whether other major cell types implicated in atherosclerosis, such as endothelial cells and macrophages, show the same response to DSBs accumulation as VSMCs, and also whether the aforementioned DNA sensors interdependently regulate innate immune responses. Collectively, these findings suggest that activation of DNA sensors by the dislocated self-DNA plays a pivotal role in atherosclerosis.

We used Ku80-deficient models to study the association between DSBs and atherosclerosis. *Ku80*-null mice have been reported to show growth retardation and defects in lymphoid organ development, while *Ku80*^+/−^ mice have been described as indistinguishable from WT mice^26^. However, *Ku80*^+/−^ mice have apparently displayed accelerated aging in skeletal muscle^51^. Together with Ku70 and DNA-dependent protein kinase catalytic subunit (DNA-PKcs), Ku80 is well characterized as a component of the DNA-PK complex, which is essential to the NHEJ repair pathway^52^. Since NHEJ is a template-independent repair system, it is presumably the primary DSB repair pathway in quiescent VSMCs. Indeed, we observed that DSBs accumulation was enhanced in both the Ku80-deficient aortas and VSMCs (Figs. 2B,C, and 3A,B), and ATM was persistently activated (Fig. 3B). Of note, *Atm* silencing failed to suppress the increase of IL-6 mRNA level in Ku80-deficient VSMCs (Fig. 4C). We only tested a transient effect of *Atm* silencing, and therefore there is still a possibility that ATM is involved in proinflammatory activation. Transient silencing of *Cgas* or *Sting*, however, was sufficient to suppress the increase of IL-6 mRNA level in Ku80-deficient VSMCs (Fig. 5E). Recent reports have indicated that the DNA-PK complex may function as a DNA sensor and mediate innate immunity^53–55^. In our study, transient silencing of the Ku80 gene revealed that Ku80 itself does not function as a signaling molecule for increased inflammatory cytokine expression (data not shown). This implies that DSBs accumulation due to Ku80 deficiency increased the cytosolic DNA fragments, resulting in activation of the cGAS-STING axis, and thereby upregulating inflammatory cytokine expression. However, in order to confirm the role of Ku80 in proinflammatory activation and draw direct conclusions, we need to study on a separation-of-function mutant, which is currently unavailable in mammalian models. Such mutant would be useful to explore the roles of Ku80 beyond DNA repair in future studies.

DNA fragments excluded from the nucleus, particularly referred to as micronuclei have long been known as one of indicators of chromosomal damage. An elevated level of micronucleus was previously reported in peripheral blood lymphocytes of patients with coronary artery disease^1^. Cytosolic DNA fragments can be formed via unrepaired DSBs and/or downregulation of the nuclear lamina protein lamin B1^56,57^. We observed a substantial increase of cytosolic DNA and a decreased level of lamin B1 in Ku80-deficient VSMCs (Figs. 3G and 5A,B). Recently, cytosolic DNA generated in senescent cells has emerged as a key mediator of the senescence-associated secretory phenotype (SASP)^40,58,59^. In the current study, Ku80-deficient VSMCs showed elevated levels of senescent markers (p16^INK4A^ and SA β-gal activity). Moreover, mRNA levels of proinflammatory cytokines (IL-6, IL-8 and MCP1), which are also known as SASP components^19^, were upregulated in the Ku80-deficient cells (Fig. 4A). Recent studies have shown that SASP is promoted by the cGAS-STING signaling and cGAS is essential for maintenance of senescence^60–62^. We also demonstrated that cellular senescence partly coincided with cGAS-STING-IRF3 activation and the expression of IL-6 in senescent cells was dependent on cGAS (Figs. 5I and 6J). Moreover, DNA damage-induced cellular senescence was abolished by *cGAS* silencing (Fig. 6K). Combining our findings with those of previous reports, cytosolic DNA induced by DSBs activates the cGAS-STING-IRF3 pathway, which triggers cellular senescence and inflammatory responses.

In this study, we demonstrated that the accumulation of DSBs preceded the onset of atherosclerosis and that the resulting cytosolic DNA fragments induced inflammation by means of Ku80-deficient models. Although further investigations are necessary to be certain of the causative role of DNA damage, our findings indicate that the accumulation of DSBs contributes to the pathogenesis of atherosclerosis by promoting proinflammatory responses via the cGAS-STING-IRF3 pathway. Thus, the cGAS-STING-IRF3 axis may be a promising therapeutic target for atherosclerosis.

### Supplementary Information

Supplementary Information.

## Data Availability

The data that support the findings of this study are available from the corresponding author upon reasonable request.

## Abbreviations

ATM: Ataxia telangiectasia mutated
ApoE: Apolipoprotein E
cGAS: Cyclic GMP-AMP synthase
DDR: DNA damage response
DSBs: DNA double-strand breaks
γH2AX: Phosphorylated histone H2AX
IFNβ: Interferon beta
IL-6: Interleukin 6
IκBα: NF-kappa B inhibitor alpha
IRF3: Interferon regulatory factor 3
MCP1: Monocyte chemoattractant protein 1
NF-κB: Nuclear factor-kappa B
NHEJ: Non-homologous end joining
SA β-gal: Senescence-associated beta-galactosidase
SASP: Senescence-associated secretory phenotype
STING: Stimulator of interferon genes
TBK1: TANK binding kinase 1
VSMCs: Vascular smooth muscle cells
