# Supplementary material for: A possible role for proinflammatory activation via cGAS-STING pathway in atherosclerosis induced by accumulation of DNA double-strand breaks
Source: Sci Rep. 2023 Sep 30;13:16470. doi: 10.1038/s41598-023-43848-7 (PMC10542807; doi:10.1038/s41598-023-43848-7)
Supplement: Supplementary file 1 — Supplementary Information. [file 41598_2023_43848_MOESM1_ESM.docx]

**SUPPLEMENTAL MATERIALS**

**A possible Role for Proinflammatory Activation via cGAS-STING Pathway in Atherosclerosis Induced by Accumulation of DNA Double-Strand Breaks**

Chiemi Sakai^1^, Keitaro Ueda^1^, Kohei Goda^1^, Rikuto Fujita^2^, Junji Maeda^3^, Shinya Nakayama^4^, Yusuke Sotomaru^5^, Satoshi Tashiro^4^, Masao Yoshizumi^1^, Takafumi Ishida^6^, *Mari Ishida^1^

1) Department of Cardiovascular Physiology and Medicine, Graduate School of Biomedical and Health Sciences, Hiroshima University

2) National Hospital Organization, Higashihiroshima Medical Center

3) Department of Cardiology, Tsuchiya General Hospital

4) Department of Cellular Biology, Research Institute for Radiation Biology and Medicine, Hiroshima University

5) Natural Science Center for Basic Research and Development, Hiroshima University

6) Department of Cardiovascular Medicine, Fukushima Medical University

Correspondence to: Mari Ishida, M.D., Ph.D., Department of Cardiovascular Physiology and Medicine, Graduate School of Biomedical and Health Sciences, Hiroshima University, 1-2-3 Kasumi, Minami-ku, Hiroshima City, Hiroshima 734-8551, Japan, Email: [mari@hiroshima-u.ac.jp](mailto:mari@hiroshima-u.ac.jp), Phone: +81822575122.

**Supplemental Methods**

**Measurement of serum and secreted IL-6 by vascular smooth muscle cells (VSMCs)**

The serum was obtained from male mice fed with a high-fat diet for 2 weeks starting from 60 days of age. The conditioned media from murine vascular smooth muscle cells were collected. IL-6 concentration was determined using mouse IL-6 Quantikine™ ELISA kit (M6000B by R&D systems, MN, USA) following manufactures protocol. Briefly, 50 µL of samples and/or standards were incubated on a ELISA plate for 2 hours at room temperature, washed with wash buffer, and incubated with 100 µL of Mouse IL-6 Conjugated for 2 hours at room temperature. After washing the plate with wash buffer, 100 µL of Stop Solution was added and the optical density of each well containing samples or standards was measured by Varioskan Flash microplate reader (Thermo Scientific) at 450 nm. Wavelength correction was performed by subtracting readings at 540 nm. IL-6 concentration was calculated from the mean absorbance of duplicate samples and standards.

**Immunofluorescent staining of aortic tissue sections**

Paraffin-embedded mice aortic arch was cross sectioned. The tissue sections were deparaffinized in xylene. Heat-induced epitope retrieval was performed on these sections at 97 ºC for 45 minutes. Serial sections were incubated with blocking buffer (1% BSA in PBST) for 1 h at a room temperature, and then incubated with anti-phosphorylated histone H2AX (γH2AX) (ser139), anti-p16^INK4A^ or anti-α smooth muscle actin (αSMA) antibodies overnight. On the following day sections were incubated with secondary antibodies conjugated with Cy3 or FITC for 2 h at a room temperature. Sections were counterstained with Hoechst 33342 (Dojindo, Kumamoto, Japan). Fluorescent images were captured with BZ-X700 (KEYENCE, Osaka, Japan).

**NF-κB nuclear translocation analysis**

Nuclear and cytoplasmic fractions were collected using NE-PER Nuclear and Cytoplasmic Extraction Reagents (Thermo Scientific). Western blotting was performed according to the method described in the main text.

**Gene silencing of NF-κB p65**

Cells were transfected with short interfering RNA (siRNA) against mouse *Rela* (s72857, ambion®, ThermoFisher), mouse *Nfkb1* (s70542, ambion®, ThermoFisher) or a negative control cocktail (AllStars Neg. Control siRNA, QIAGEN) using Lipofectamine™ RNAiMAX Transfection Reagent (ThermoFisher) 48–72 hours prior to harvesting samples.

**Supplemental Figure 1**

**
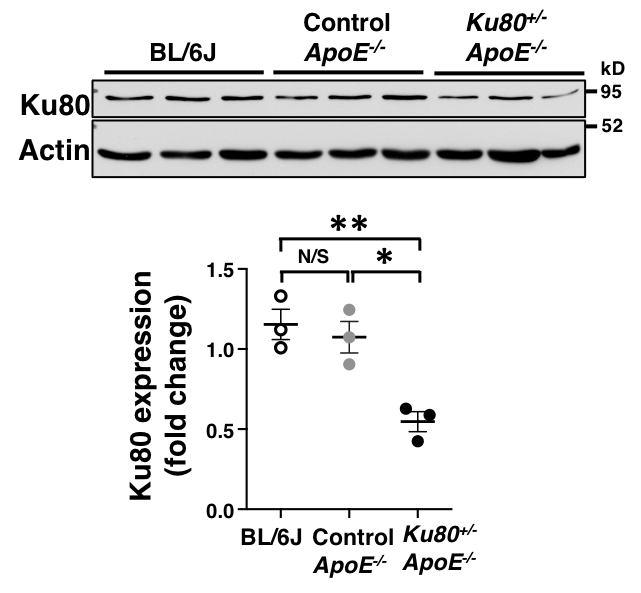
**

**Supplemental Figure 1.** Ku80 protein expression in the aortas of mice. Quantification in the bottom (n = 3 for each group). **p* < 0.05, ***p* < 0.01, N/S: not significant. *p*-value was determined by one-way ANOVA. Data are expressed as mean ± SEM. Bands are cropped from the original blots presented in Supplemental Figure 12. ­­­

**Supplemental Figure 2**

**
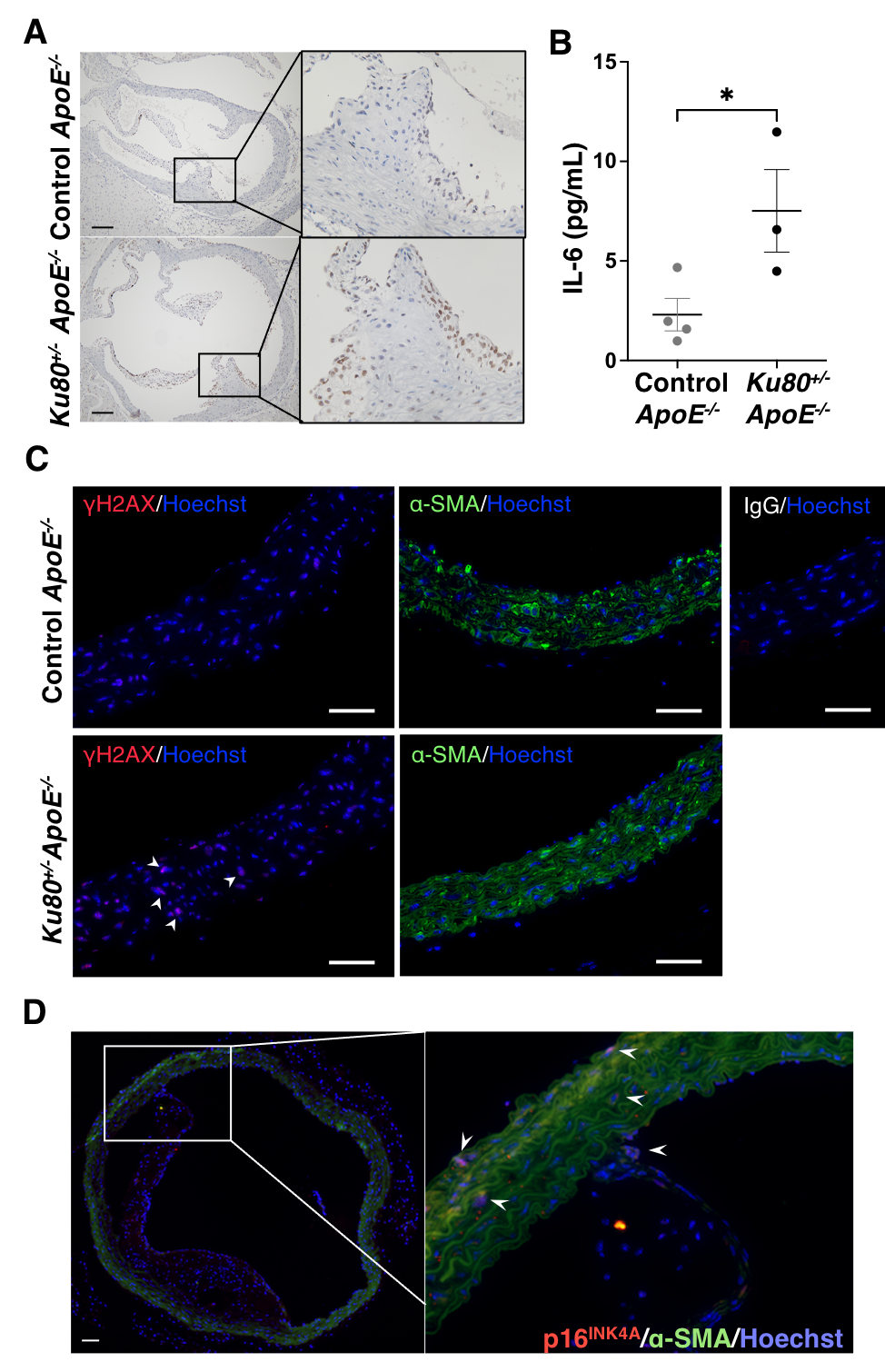
**

**Supplemental Figure 2**. A) Representative images of the aortic valve. The sections were immunohistochemically stained for γH2AX. Bar = 100 µm. B) IL-6 concentration in serum of mice fed on a high-fat diet for 2 weeks (Control *ApoE^-/-^*; n = 4, *Ku80^+/-^ ApoE^-/-^*; n = 3). **p* < 0.05. *p*-value was determined by unpaired t-test. Data are expressed as mean ± SEM. C) Immunofluorescent images of the aortas obtained from the mice fed on a high-fat diet for 2 weeks. The serial tissue sections were stained with antibodies against γH2AX, α-smooth muscle actin (α-SMA) or incubated with normal IgG for the negative control. The white arrowheads represent γH2AX-positive smooth muscle cells. γH2AX in red; α-SMA in green; the nuclei stained with Hoechst 33342 in blue. Bar = 50 µm. D) Immunofluorescent detection of p16^INK4A^-positive senescent cells in the *Ku80^+/-^/ApoE^-/-^* aorta after 4 weeks of high-fat diet feeding. The white arrowheads represent p16^INK4A^-positive smooth muscle cells. p16^INK4A^ in red; α-SMA in green; the nuclei stained with Hoechst 33342 in blue. Bar = 50 µm.

**Supplemental Figure 3**

**
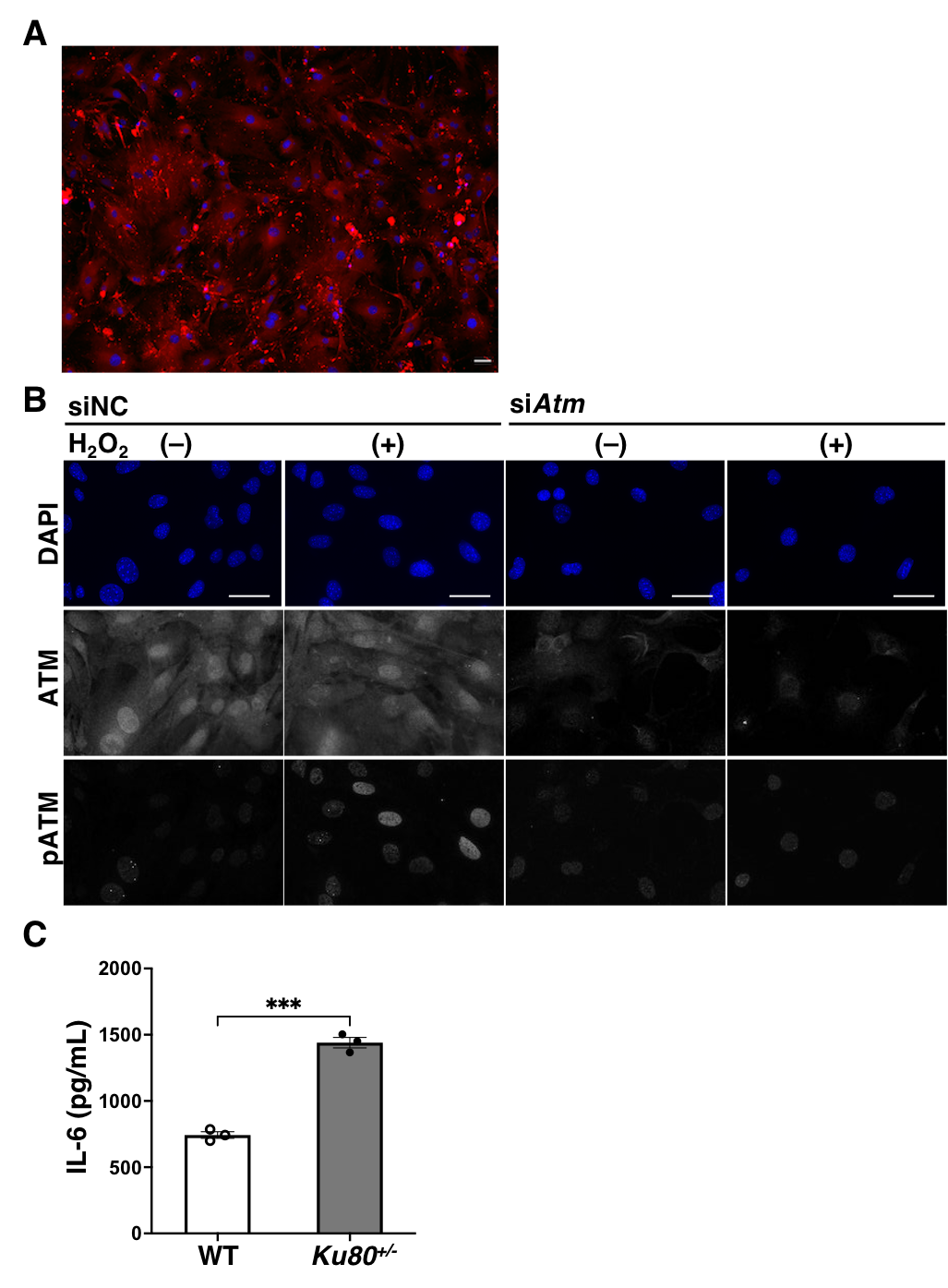
**

**Supplemental Figure 3.** A) Representative image of vascular smooth muscle cells (VSMCs) isolated from *Ku80^+/-^* mouse. The cells were immunostained with α-SMA antibody (in red) and the nuclei were counterstained with DAPI (in blue). Bar = 20 µm. B) Immunofluorescent staining of total and phosphorylated ATM in ATM-silenced VSMCs exposed to hydrogen peroxide (100 µM) for 2 h. ATM in green; pATM in red: the nuclei stained with DAPI in blue Bar = 50 µm. C) Concentration of IL-6 secreted from murine VSMCs at a basal level (n = 3 each). ****p* < 0.0001. *p*-value was determined by unpaired t-test. Data are expressed as mean ± SEM.

**Supplemental Figure 4**

**
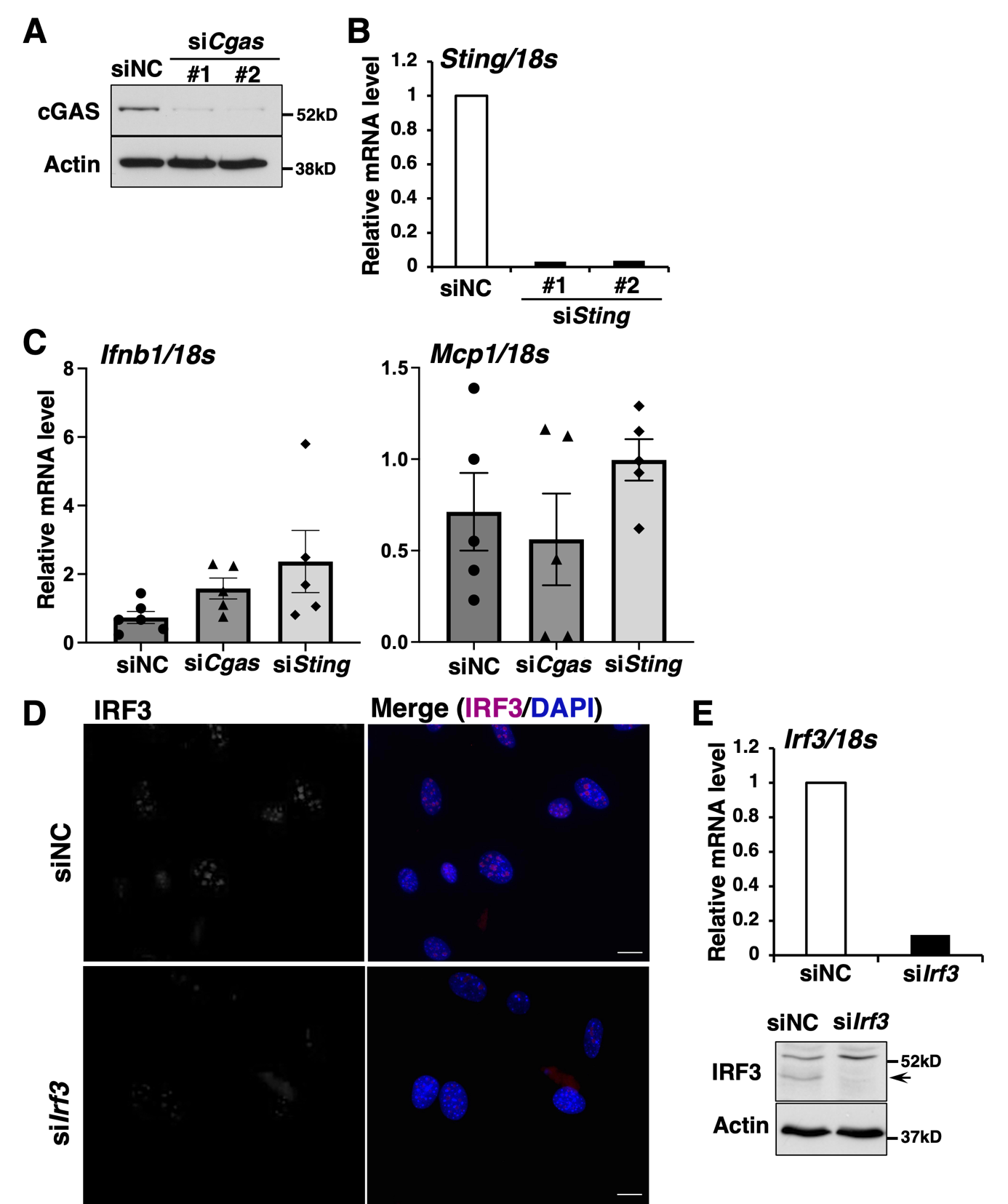
**

**Supplemental Figure 4.** A) *Ku80^+/-^* VSMCs were transfected with siRNA against *Cgas.* Western blot analysis of cGAS expression in whole cell lysates from *Cgas*-silenced cells. B) *Ku80^+/-^* VSMCs were transfected with siRNA against *Sting*. *Sting*-silencing was assessed by real-time qPCR analysis. C) real-time qPCR analysis of IFNb1 (left) and MCP1(right) mRNA expression in *Ku80^+/-^* cells received either *Cgas* or *Sting*-silencing treatment (n = 5). D) Immunofluorescent staining of IRF3 in *Ku80^+/-^* VSMCs with or without *Irf3* silencing. IRF3 in red; the nuclei stained with DAPI in blue. Bar = 20 µm. E) *Irf3*-silencing in *Ku80^+/-^* VSMCs assessed by real-time qPCR (top). Western blot analysis of IRF3 to confirm IRF3 depletion in the cells (bottom). Bands are cropped from the original blots presented in Supplemental Figure 13. Data are expressed as mean ± SEM.

**Supplemental Figure 5**

**
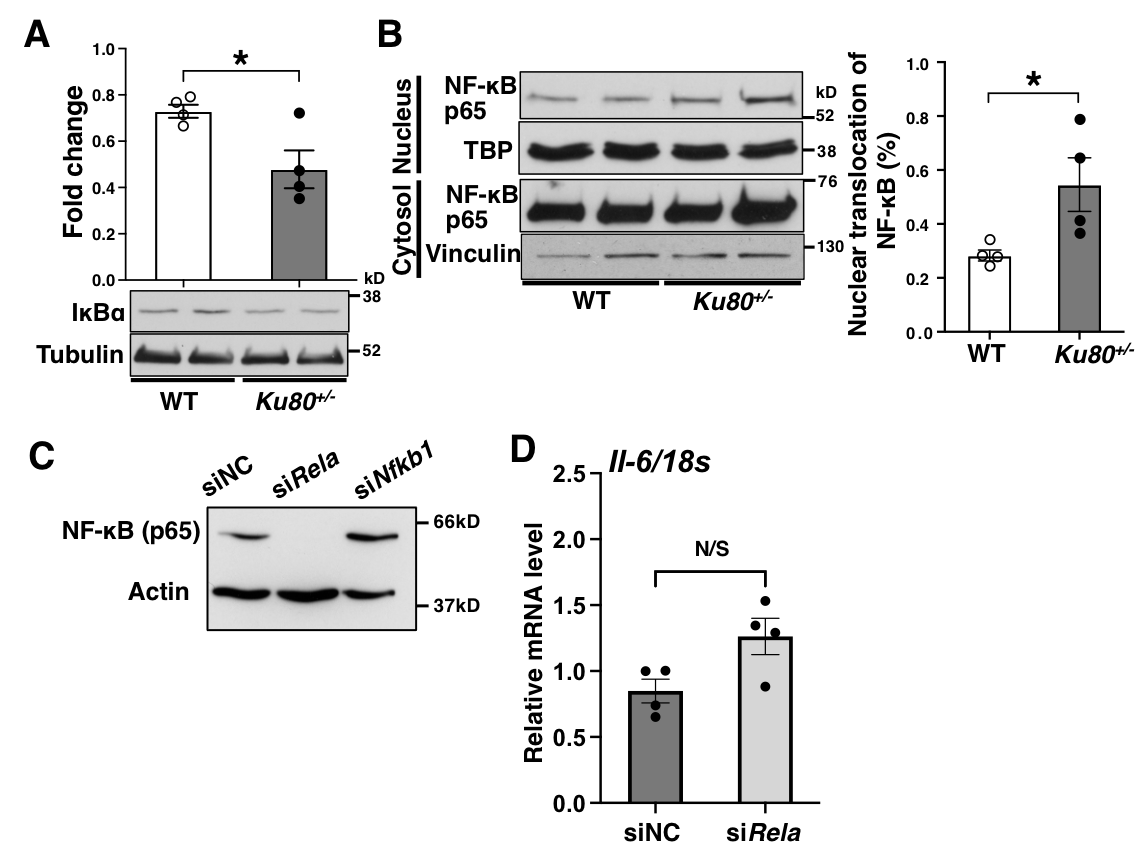
**

**Supplemental Figure 5.** Western blot analysis of A) IκBα expression (n = 4), B) NF-κB nuclear translocation (n = 4) in murine VSMCs. **p* < 0.05. C) NF-κB p65 (*Rela*) was silenced in *Ku80^+/-^* VSMCs. NF-κB p105 (*Nfkb1*) was silenced at the same time to confirm the siRNA specificity. NF-κB expression was detected by western blotting. D) mRNA levels of IL-6 in NF-κB p65-silenced *Ku80^+/-^* VSMCs were determined by real-time qPCR. N/S: not significant. *p*-value was determined using unpaired *t*-test. Data are expressed as mean ± SEM. Bands are cropped from the original blots presented in Supplemental Figure 14.

**Supplemental Figure 6**

**
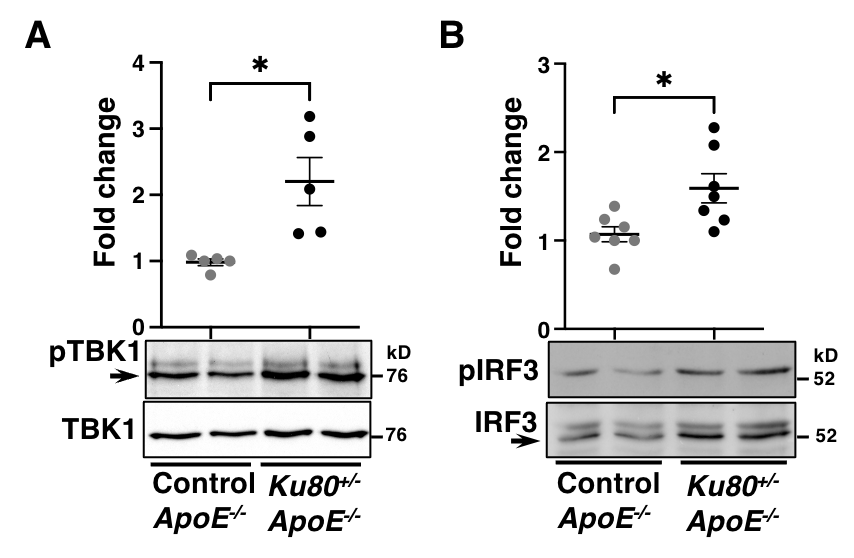
**

**Supplemental Figure 6.** Western blot analysis of A)TANK-binding protein kinase 1(TBK1) phosphorylation (Ser172) (n = 5) and B) interferon regulatory factor 3 (IRF3) phosphorylation (Ser396) (n = 7) in the aortic tissue lysates from mice fed on a high-fat diet for 2 weeks. Target bands were indicated by the arrow. **p* < 0.05. *p*-value was determined using unpaired *t*-test. Data are expressed as mean ± SEM. Bands are cropped from the original blots presented in Supplemental Figure 15.

**Supplemental Figure 7**


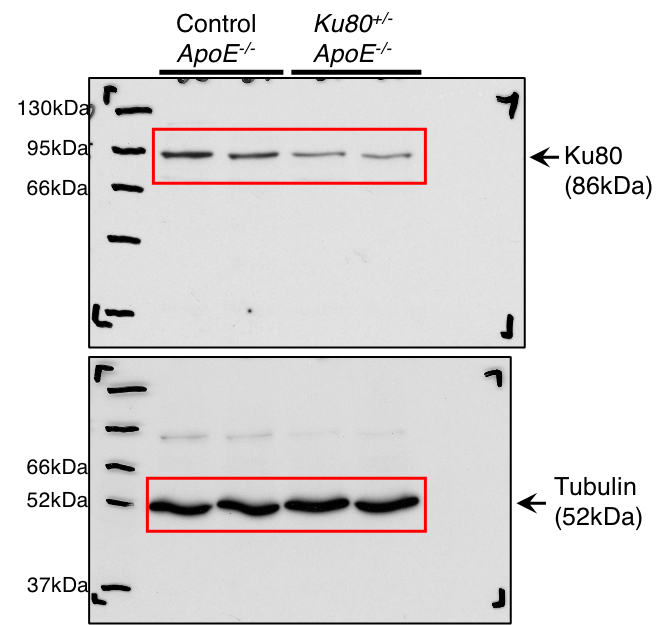


**Supplemental Figure 7.** Original western blots of Ku80. Ku80 and tubulin was detected on the same gel. Images in the red rectangles are shown in Figure 1B.

**Supplemental Figure 8**


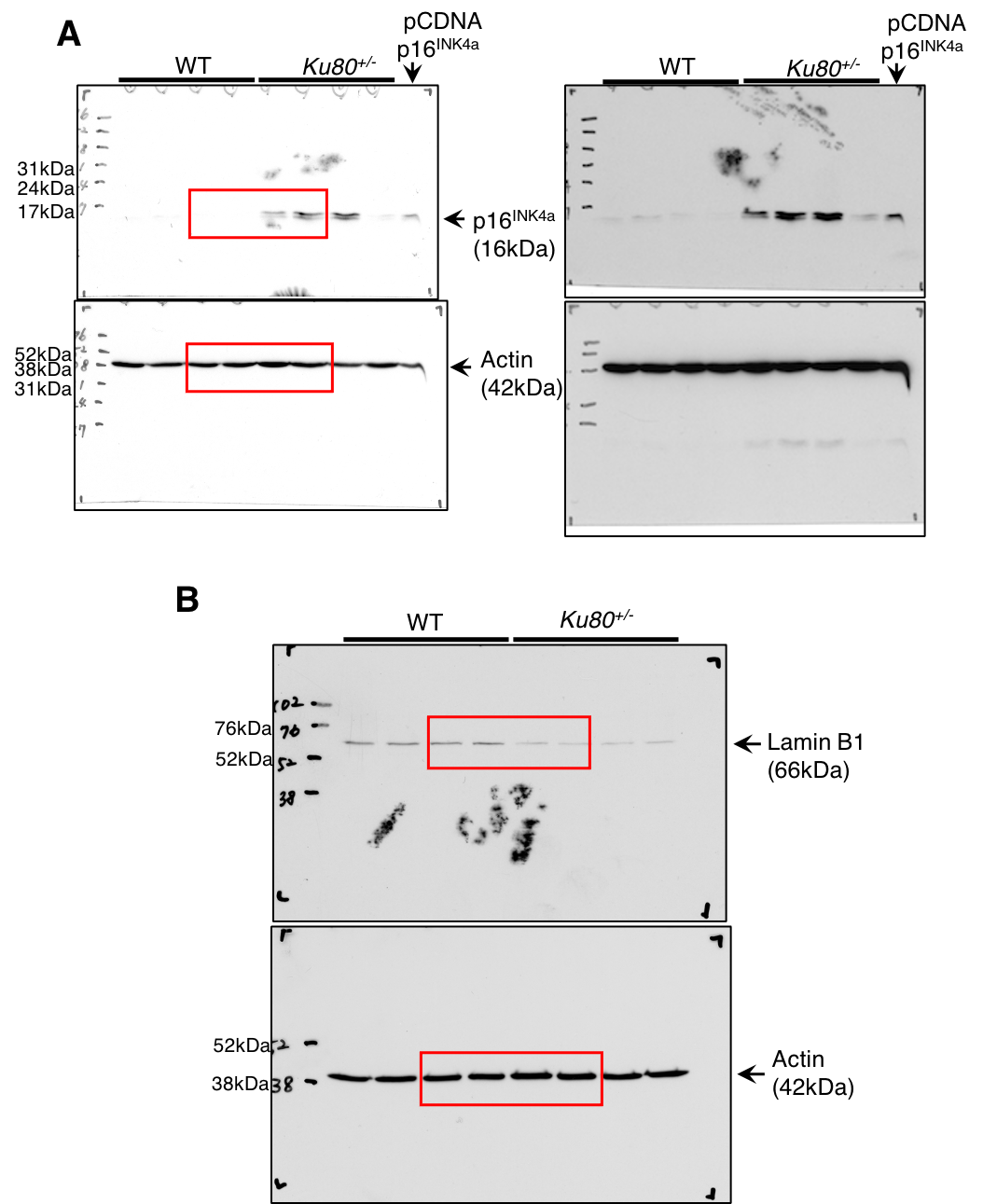


**Supplemental Figure 8.** Original western blots of A) p16^INK4A^ and B) Lamin B1. A) pCDNA p16^INK4A^: wild type cells transfected with pCDNA p16^INK4A^ as positive control. p16^INK4A^ and actin were detected on the same gel. Images on the right: longer exposure. B) Lamin B1 and actin were detected on the same gel. Images in the red rectangles are shown in Figure 3F and 3G.

**Supplemental Figure 9**


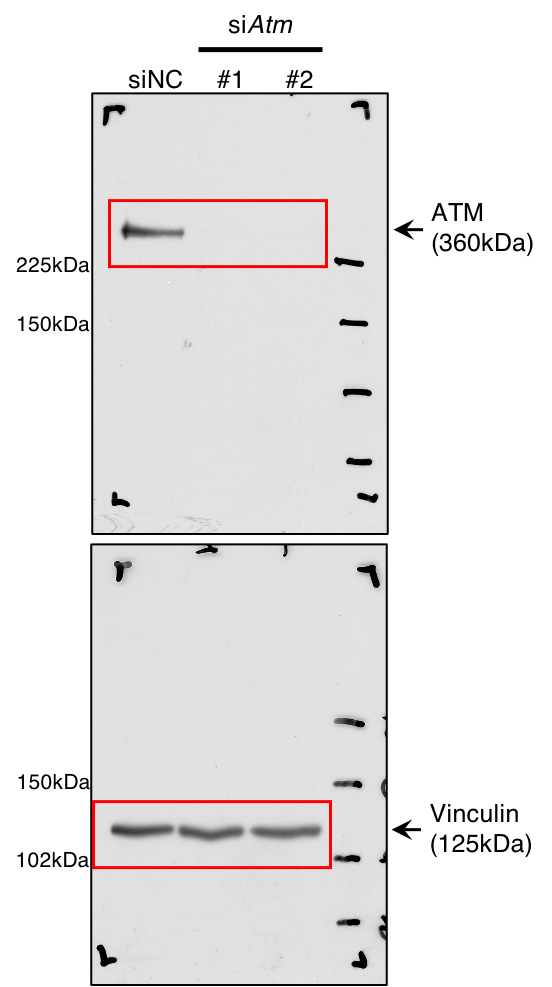


**Supplemental Figure 9.** Original western blots of ATM. Wild type cells were transfected with either negative control siRNA (siNC) or siRNA against *Atm* (si*Atm*#1 or si*Atm*#2). ATM and vinculin were detected on the same gel. Images in the red rectangles are shown in Figure 4B.

**Supplemental Figure 10**


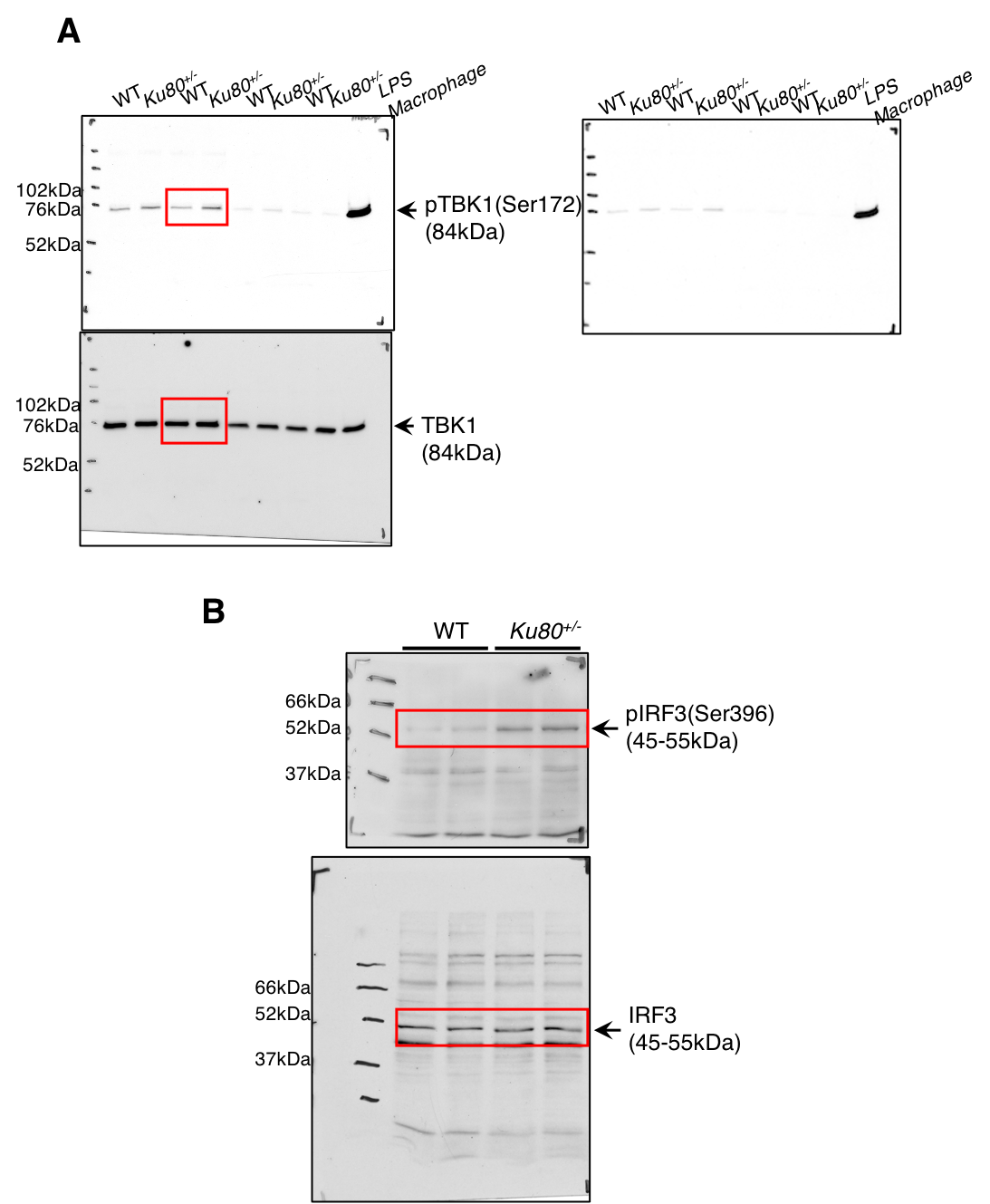


**Supplemental Figure 10.** Original western blots of A) phosphorylated TBK1 and B) IRF3. A) phosphorylated TBK1(pTBK1) and total TBK1 were detected on the same gel. The image on the right: shorter exposure. B) pIRF and total IRF3 were detected on separate gels run at the same time. Images in the red rectangles are shown in Figure 5D and 5F.

**Supplemental Figure 11**

**
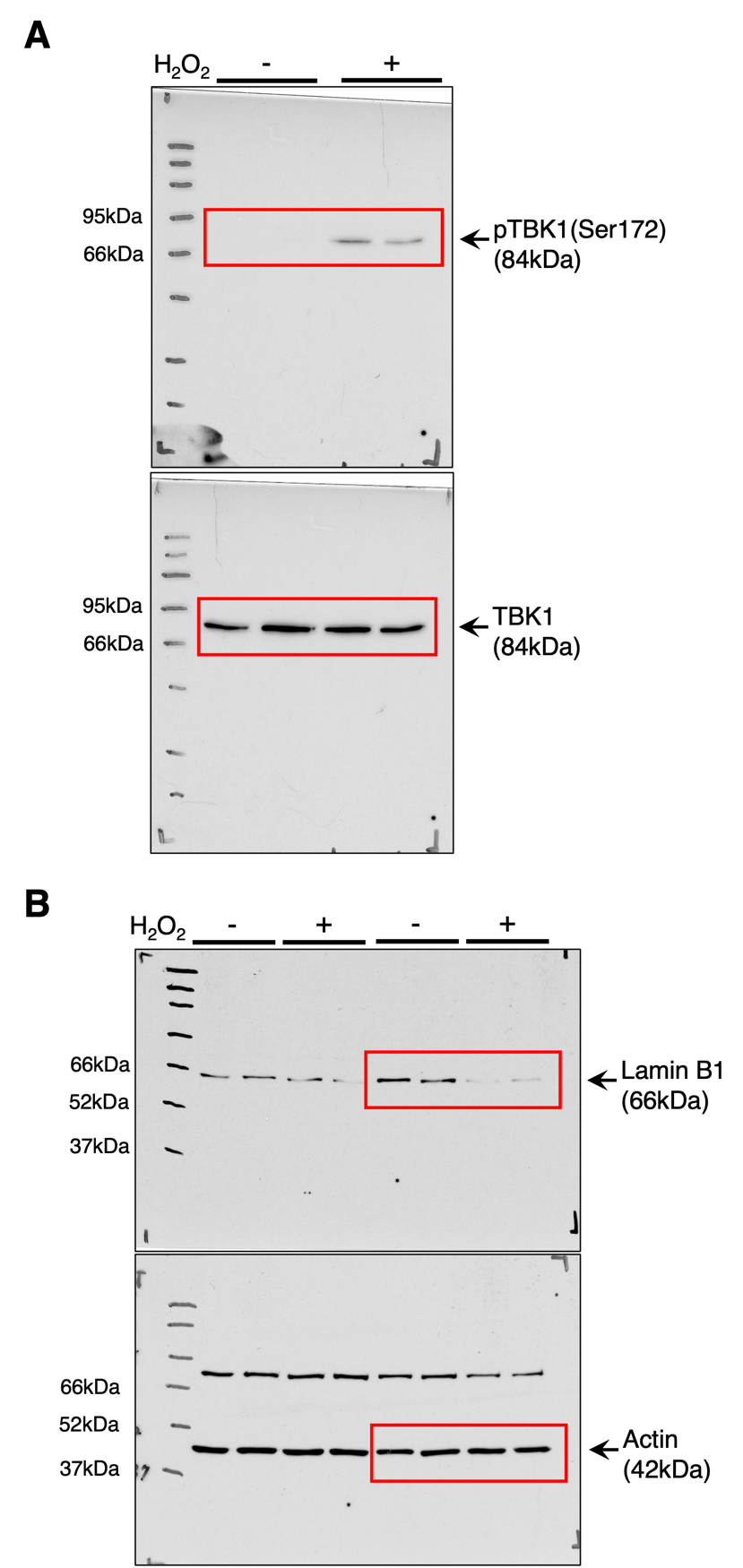
**

**Supplemental Figure 11.** Original western blots of A) phosphorylated TBK1 (Ser 172) and B) lamin B1. A) Phosphorylated TBK1 and total TBK1 were detected on the same gel. B) Lamin B1 and actin were detected on the same gel. Images in the red rectangles are shown in Figure 6D and 6H.

**Supplemental Figure 12**


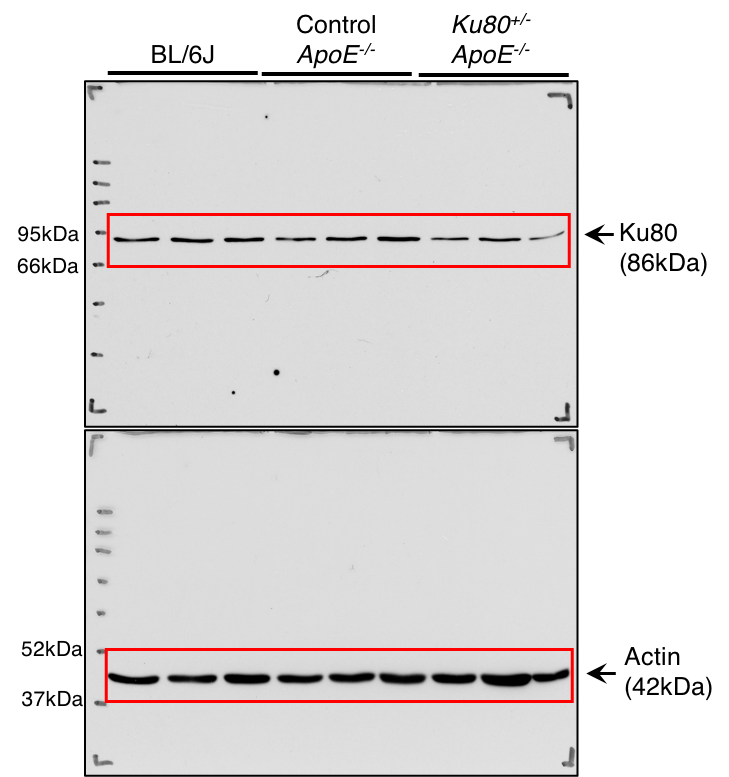


**Supplemental Figure 12.** Original western blots of Ku80. Ku80 and actin were detected on the same gel. Images in the red rectangles are shown in Supplemental Figure 1.

**Supplemental Figure 13**


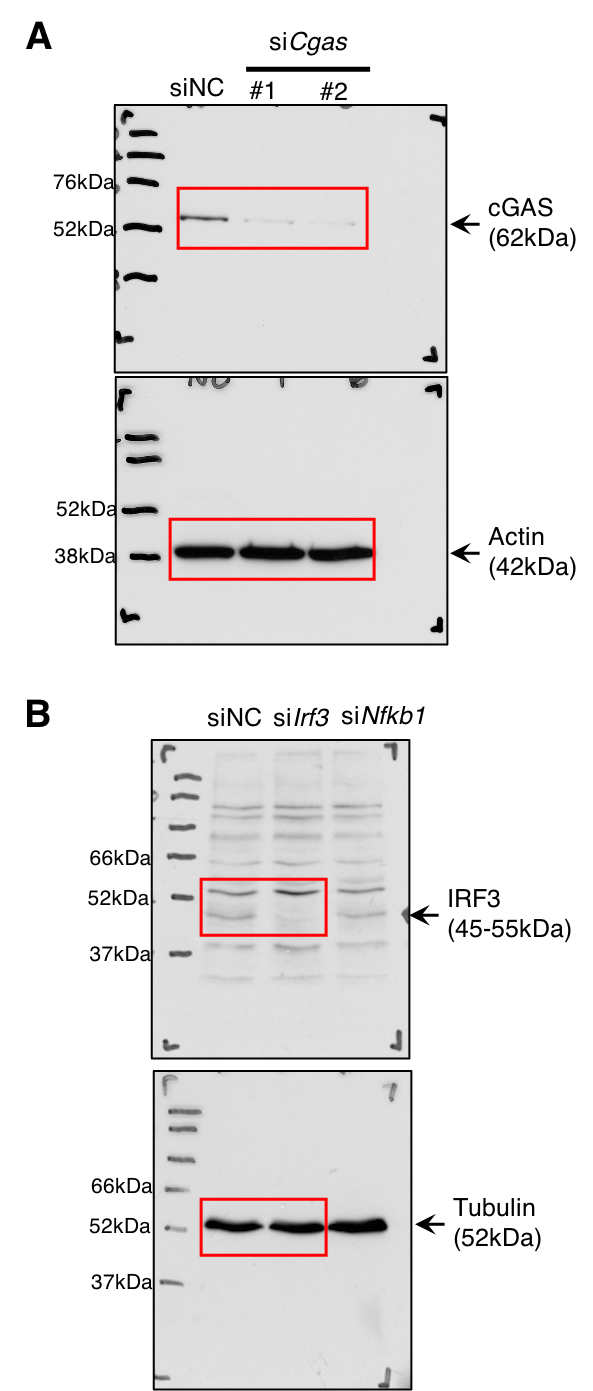


**Supplemental Figure 13.** Original western blots of A) cGAS and B) IRF3. A) cGAS and actin were detected on the same gel. B) IRF3 and tubulin were detected on the same gel. Images in the red rectangles are shown in Supplemental Figure 4A and 4D.

**Supplemental Figure 14**

**
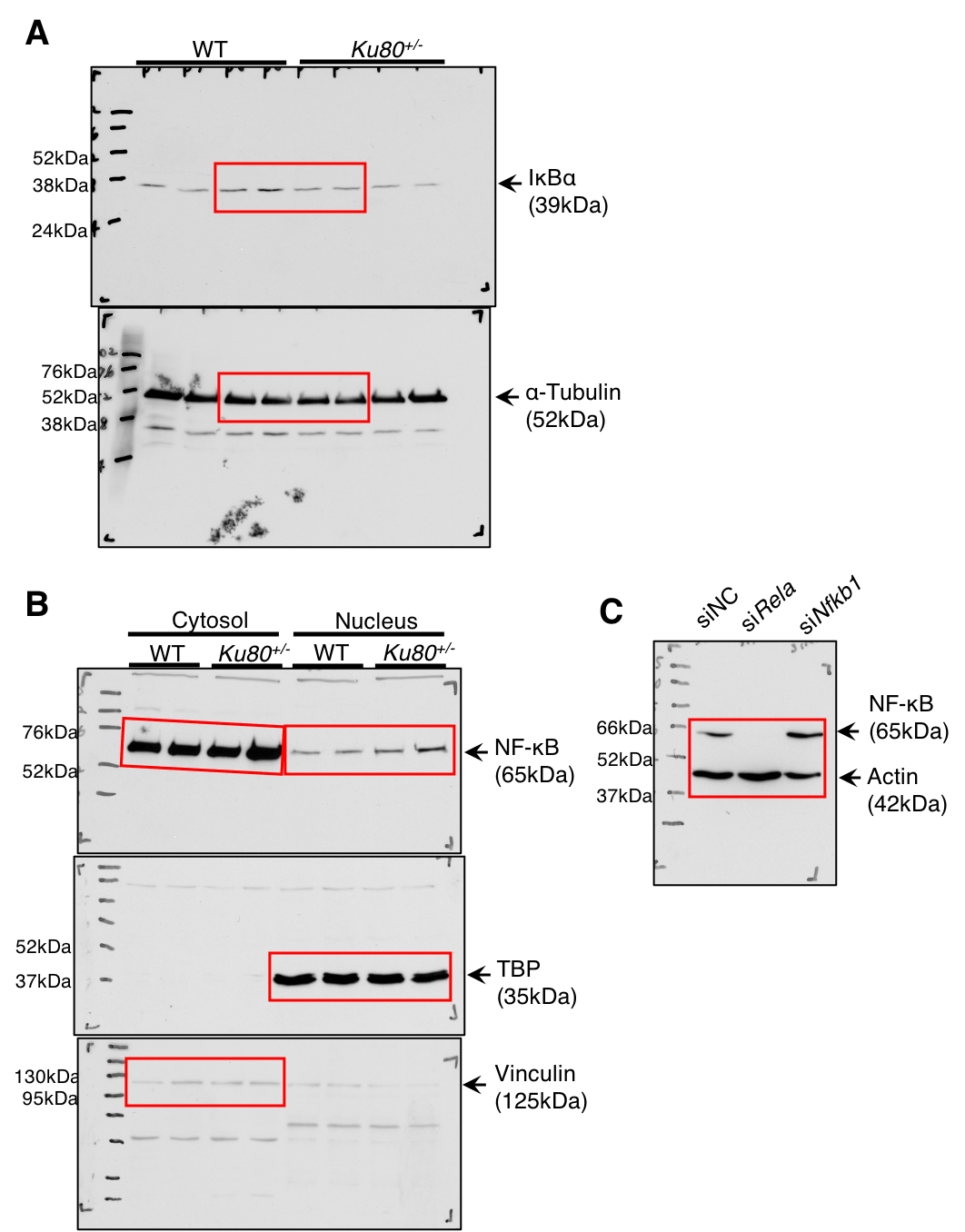
**

**Supplemental Figure 14.** Original western blots of A) IκBα, B) and C) NF-κB. A) IκBα and α-Tubulin were detected on the same gel. B) NF-κB and TBP were detected on the same gel. The same samples were loaded on a different gel to detect Vinculin. C) NF-κB and actin were detected at the same time. Images in the red rectangles are shown in Supplemental Figure 5A, 5B and 5C.

**Supplemental Figure 15**

**
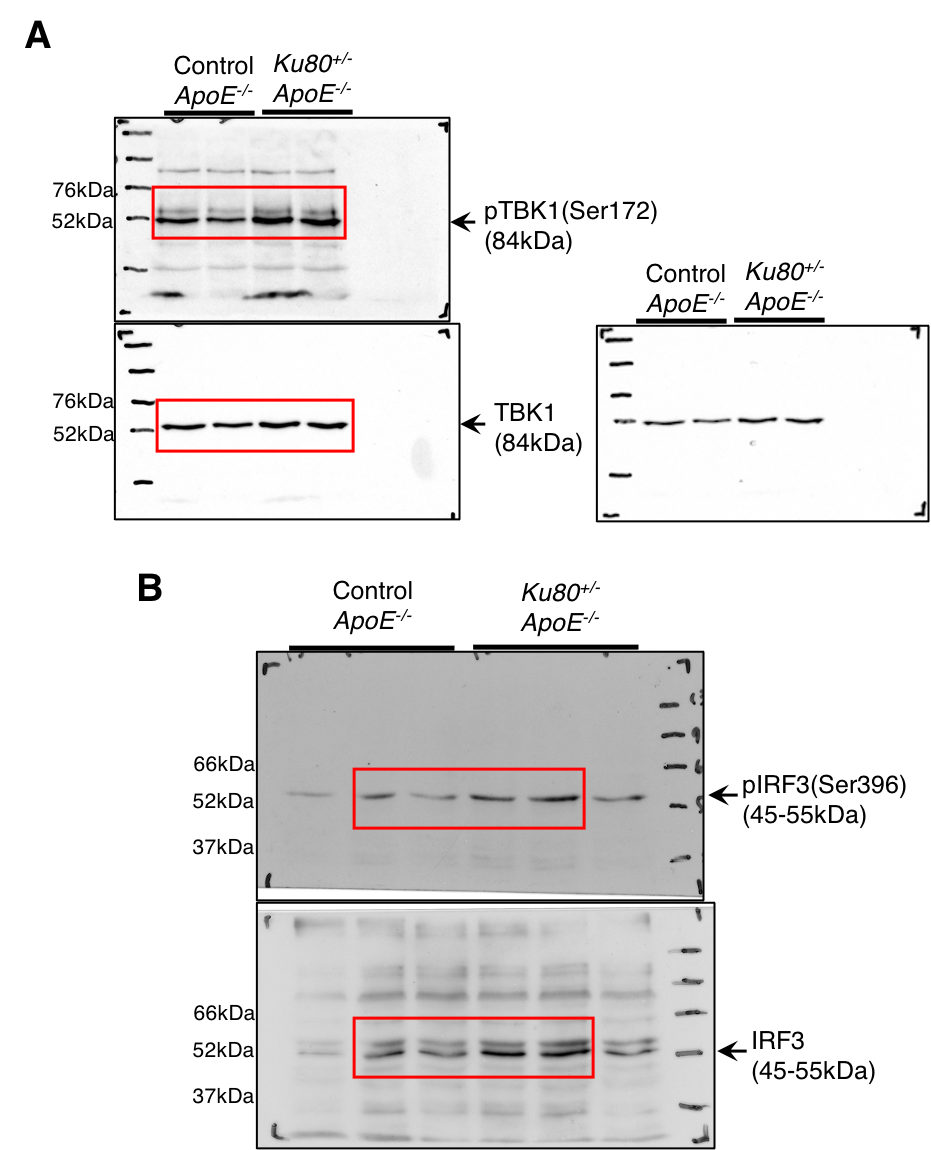
**

**Supplemental Figure 15.** Original western blots of A) phosphorylated TBK1 and B) IRF3. A) phosphorylated TBK1 and total TBK1 were detected on the same gel. The image on the right: shorter exposure. B) phosphorylated IRF3 and total IRF3 were detected on the same gel. Images in the red rectangles are shown in Supplemental Figure 6A and 6B.

**Supplemental Table 1. Sequences of the primers used in the real-time PCR analysis**.

| Name | 5’-3’ sequence | |
| --- | --- | --- |
|  | Forward primer | Reverse primer |
| *Il1b* | TCCAGGATGAGGACATGAGCAC | GAACGTCACACACCAGCAGGTTA |
| *Il6* | CAACGATGATGCACTTGCAGA | CTCCAGGTAGCTATGGTACTCCAGA |
| *Mcp1* | AGCAGCAGGTGTCCCAAAGA | GTGCTGAAGACCTTAGGGCAGA |
| *Il8* | CTCCTGCTGGCTGTCCTTAAC | CCTGAATACACAGACATCGTAGCTC |
| *Angptl2* | GGAGGTTGGACTGTCATCCAGAG | GCCTTGGTTCGTCAGCCAGTA |
| *p16ink4a* | GAACTCTTTCGGTCGTACCC | CGAATCTGCACCGTAGTTGA |
| *Ifnb1* | CAGCTCCAAGAAAGGACGAAC | GGCAGTGTAACTCTTCTGCAT |
| *B2m* | ACAGTTCCACCCGCCTCACATT | TAGAAAGACCAGTCCTTGCTGAAG |
| *Irf3* | CGGAAAGAAGTGTTGCGGTTAGC | CAGGCTGCTTTTGCCATTGGTG |
| 18s rRNA | GAATCGAACCCTGATTCCCCGTC | CGGCGACGACCCATTCGAAC |

**Supplemental Table 2. List of antibodies used in western blotting and immunofluorescent analyses**.

| **Target antigen** | **Vendor** | **Catalog #** | **Working concentration** |
| --- | --- | --- | --- |
| Ku80 | Cell Signaling Technology | 2753 | 1:1000 |
| γH2AX (Ser139) | abcam | ab124781 | 1:500 |
| pATM (Ser1981) | Rockland | 200-301-400 | 1:500 |
| ATM | Cell Signaling Technology | 2873 | 1:1000 |
| p16^INK4a^ | abcam | ab211542 | 1:2000 |
| Lamin B1 | Proteintech | 66095-1-Ig | 1:10000 |
| pTBK1 (Ser172) | Cell Signaling Technology | 5483 | 1:1000 |
| TBK1 | Cell Signaling Technology | 3504 | 1:1000 |
| NF-κB | Cell Signaling Technology | 8242 | 1:1000 |
| IκBα | Cell Signaling Technology | 4812 | 1:1000 |
| pIRF3 | Cell Signaling Technology | 4947 | 1:1000 |
| IRF3 | Proteintech | 11312-1-AP | 1:10000 |
| αSMA | Sigma-Aldrich | A2547 | 1:100 |
| cGAS | Cell Signaling Technology | 31659 | 1:1000 |
| Actin | Sigma-Aldrich | A2066 | 1:1000 |
| Vinculin | Sigma-Aldrich | V4505 | 1:500 |
| TBP | abcam | ab818 | 1:2000 |
